# Roles of transcriptional factor PsrA in the regulation of quorum sensing in *Pseudomonas aeruginosa* PAO1

**DOI:** 10.3389/fmicb.2024.1424330

**Published:** 2024-06-26

**Authors:** Li-Ching Kok, Chia-Chun Tsai, Yu-Hsuan Liao, Yi-Ling Lo, Nai-Wei Cheng, Ching-Ting Lin, Hwan-You Chang

**Affiliations:** ^1^Institute of Molecular Medicine, National Tsing Hua University, Hsinchu City, Taiwan; ^2^School of Chinese Medicine, China Medical University, Taichung City, Taiwan

**Keywords:** fatty acid, gene expression, LASR, oleic acid, *Pseudomonas aeruginosa*, PSRA, quorum sensing, transcription factor

## Abstract

The transcription factor PsrA regulates fatty acid metabolism, the type III secretion system, and quinolone signaling quorum sensing system in *Pseudomonas aeruginosa*. To explore additional roles of PsrA in *P. aeruginosa*, this study engineered a *P. aeruginosa* PAO1 strain to carry a recombinant plasmid with the *psrA* gene (pMMB*psrA*) and examined the impact of elevated *psrA* expression to the bacterium. Transcriptomic analysis revealed that PsrA significantly downregulated genes encoding the master quorum-sensing regulators, RhlR and LasR, and influenced many quorum-sensing-associated genes. The role of PsrA in quorum sensing was further corroborated by testing autoinducer synthesis in PAO1 [pMMB*psrA*] using two reporter bacteria strains *Chromobacterium violaceum* CV026 and *Escherichia coli* [pSB1075], which respond to short- and long-chain acyl homoserine lactones, respectively. Phenotypic comparisons of isogenic Δ*psrA*, Δ*lasR*, and Δ*psrA*Δ*lasR* mutants revealed that the reduced elastase, caseinase, and swarming activity in PAO1 [pMMB*psrA*] were likely mediated through LasR. Additionally, electrophoretic mobility shift assays demonstrated that recombinant PsrA could bind to the *lasR* promoter at a 5’-AAACGTTTGCTT-3′ sequence, which displays moderate similarity to the previously reported consensus PsrA binding motif. Furthermore, the PsrA effector molecule oleic acid inhibited PsrA binding to the *lasR* promoter and restored several quorum sensing-related phenotypes to wild-type levels. These findings suggest that PsrA regulates certain quorum-sensing phenotypes by negatively regulating *lasR* expression, with oleic acid acting as a crucial signaling molecule.

## 1 Introduction

*Pseudomonas aeruginosa* is a Gram-negative, rod-shaped pathogen that primarily infects immunocompromised individuals and cystic fibrosis patients (Rowe et al., 2005). The bacterium is notorious for its multidrug resistance and biofilm formation, which often complicates treatment (Livermore, 2002). In addition to delivering proteins into host cells via the type III secretion system, *P. aeruginosa* produces a plethora of pathogenicity factors, including various adhesins and secreted toxins such as exotoxin A, elastases, and hemolysins (Wick et al., 1990; Hong and Ghebrehiwet, 1992; Van Delden and Iglewski, 1998; Kipnis et al., 2006). In addition, most *P. aeruginosa* strains produce pyocyanin, a redox-active blue-green phenazine pigment, which also acts as a terminal signal in the quorum-sensing systems in *P. aeruginosa* (Dietrich et al., 2006). This pigment affects the respiratory epithelium by disrupting electron transport and metabolism (Denning et al., 1998; Rada and Leto, 2013), and is crucial in *P. aeruginosa*-mediated pathogenesis in a mouse lung infection model of cystic fibrosis (Lau et al., 2004b).

The expression of many virulence genes in *P. aeruginosa* is governed by a complex cell-density responding mechanism named quorum sensing (Smith and Iglewski, 2003; Jimenez et al., 2012). Three major quorum-sensing systems have been well characterized in *P. aeruginosa*: the Lux family Las and Rhl systems, which use acyl-homoserine lactone signals, and the PQS (Pseudomonas Quinolone Signal) system, which use 2-alkyl-4-quinolones as its cognate signals. The transcription factor LasR is considered the master regulator of quorum sensing. In concert with the autoinducer *N*-3-oxo-dodecanoyl-l-homoserine lactone (3-oxo-C_12_-HSL) synthesized by synthase LasI, LasR coordinates the expression of other quorum sensing regulators and many virulence genes, including *rhlR* (Schuster and Greenberg, 2006), *pqsR* (Schuster and Greenberg, 2006), *vqsR* (Li et al., 2007), *lasB* (elastase) (Gambello and Iglewski, 1991), *lasA* (elastase) (Toder et al., 1991; Passador et al., 1993), *aprA* (alkaline protease) (Gambello et al., 1993), *toxA* (exotoxin A) (Storey et al., 1998), and *phz1* and *phz2* operons, which encode enzymes involved in pyocyanin synthesis (Gallagher et al., 2002; Lau et al., 2004a). LasR is also regulated by several transcription factors including AlgR2 (Ledgham et al., 2003), GacA/GacS (Parkins et al., 2001), MvaT (Diggle et al., 2002), RpoN (Heurlier et al., 2003; Thompson et al., 2003), and Vfr (Albus et al., 1997), thus forming a complex virulence regulatory network in *P. aeruginosa*.

PsrA, a TetR family transcription factor, has been shown to bind a palindromic sequence (C/GAAACN_2–4_GTTTG/C) present in numerous promoters including its own and that of the stationary-phase sigma factor gene *rpoS* (Kojic and Venturi, 2001; Kojic et al., 2002). Multiple studies have identified PsrA as a regulator of the type III secretion system (T3SS) operon *exsCEBA* (Shen et al., 2006; Kang et al., 2009), the integron integrase gene LexA (Novovic et al., 2019), and the fatty acid catabolism operon *fadBA5* (Kojic et al., 2005; Son et al., 2007; Kang et al., 2008). Notably, specific long-chain fatty acids can relieve PsrA repression on the acyl-CoA dehydrogenase gene *fadE* (*PA0506*), leading to increased production of 2-heptyl-3-hydroxy-4-quinolone, the autoinducer of the PQS quorum-sensing system, an extracellular compound whose production peaks in the late stationary phase (Kojic et al., 2005; Wells et al., 2017). Similar roles for PsrA in regulating quorum sensing and phenazine synthesis have been observed in *Pseudomonas syringae* and *Pseudomonas chlororaphis* (Chin et al., 2005; Chatterjee et al., 2007), respectively. Collectively, these findings strongly imply PsrA’s intricate involvement in regulating various bacterial virulence factors via the quorum sensing system.

It has been demonstrated that the expression of *psrA* is induced *in vivo* in CF patients (Son et al., 2007) and *in vitro* by long-chain fatty acids (Kang et al., 2008, 2009; Wells et al., 2017), the natural quorum sensing inhibitor protoanemonin (Bobadilla Fazzini et al., 2013), and lung surfactant (LaBauve and Wargo, 2014). However, the impact of high *psrA* expression levels on *P. aeruginosa* physiology remains unclear. To provide complementary insights to studies using gene knockout strains and to replicate the condition of *psrA* upregulation, this study constructed a *psrA* overexpression clone to investigate its effect on various virulence-related properties of *P. aeruginosa* PAO1. Extensive transcriptomic and phenotypic analyses demonstrated that PsrA overexpression triggers several quorum sensing-related phenotypes in *P. aeruginosa* PAO1, most likely through direct downregulation of *lasR* gene expression.

## 2 Materials and methods

### 2.1 Bacterial strains and growth conditions

The bacterial strains and plasmids used in this study are listed in Supplementary Table S1. All bacterial strains were routinely propagated in Luria-Bertani (LB) broth (1% tryptone, 0.5% yeast extract, and 1% NaCl). The bacterial strains carrying pMMB66EH-derivative plasmids were grown in LB supplemented with 500 μg/mL carbenicillin. M8 medium for the motility assay was prepared with the following components: 24 mM Na_2_HPO_4_, 22 mM KH_2_PO_4_, 8 mM NaCl, 0.5% casamino acid, 0.4% glucose, 0.2 mM MgSO_4_, and 0.1 mM CaCl_2_. The overnight bacterial culture was prepared as follows: a single colony of each strain was inoculated from the LB agar plate to LB broth and incubated at 37°C for 18 h at 200 rpm. Subsequently, it was diluted 1:100 in fresh LB broth and incubated for 18 h at 37°C. All chemicals used in this study were reagent grade and purchased from Sigma-Aldrich unless indicated otherwise. A stock solution of sodium oleate is prepared at 200 mM.

### 2.2 Generation of deletion mutants, complementary, and overexpression strains

Single and double deletion mutants of *psrA* and *lasR* were constructed in *P. aeruginosa* PAO1 using the allelic exchange approach (Hoang et al., 1998). The genomic sequence of *P. aeruginosa* PAO1 was obtained from the Pseudomonas genome database^1^. DNA of fragments approximately 1 kb in size flanking the coding regions were amplified using PCR and cloned in tandem into the suicide vector pEX18Tc (Supplementary Table S1). The generated plasmids were transformed separately into *Escherichia coli* S17 λpir and subsequently introduced into *P. aeruginosa* by conjugation. The transconjugants were selected on LB plates containing tetracycline (100 μg/mL), and the second crossover strains were selected positively on LB plates supplemented with 10% sucrose. The generation of the *psrA* overexpression clone was performed by cloning the *psrA* coding region into EcoRI-HindIII sites of the wide-host-range, low-copy-number plasmid pMMB66EH (Fürste et al., 1986) (Supplementary Table S1). The resulting plasmid was then mobilized into *P. aeruginosa* PAO1 via conjugation. The deletion of *psrA* or *lasR* in the bacterial clones and the presence of the pMMB66EH-derived *psrA* containing complementation plasmid were confirmed by PCR (Supplementary Table S2) and DNA sequencing.

### 2.3 Determination of *P. aeruginosa* biological properties

#### 2.3.1 Pyocyanin production

The quantity of pyocyanin was determined as described (Palumbo, 1972). Briefly, two volumes of chloroform were added into the overnight bacterial culture supernatant and mixed by shaking for 1 hour. The mixture was then centrifuged, and the bottom solvent layer was acidified with 0.2 N HCl. The pyocyanin quantity was spectrophotometrically determined at 520 nm.

#### 2.3.2 Elastase activity

The elastase activity was determined by mixing ten microliters of the filtrate prepared from overnight *P. aeruginosa* culture with 990 μL of a solution consisting of 100 mM Tris–HCl (pH 7.2), 5 mg/mL elastin-Congo red, and 1 mM CaCl_2_. The reaction mixture was incubated at 37°C with gentle shaking for four hours before the terminated addition of ten microliters of 0.12 M ethylenediaminetetraacetic acid (EDTA). The dissolved Congo red was measured at 495 nm after centrifugation (Bjorn et al., 1979).

#### 2.3.3 Hemolytic activity

The hemolytic activity of different *P. aeruginosa* strains were assayed using tryptic soy agar with 5% sheep blood. Three microliters of *P. aeruginosa* overnight culture were spotted on the plates and incubated at 30°C for 24 h. The size of the clear zone that represented bacterial hemolytic activity was scored.

#### 2.3.4 Caseinase activity

Caseinase activity was determined according to the method described by Sokol et al. (1979). Three microliters of *P. aeruginosa* overnight culture were inoculated on the casein agar plates, which contained 3% (w/v) skimmed milk powder, 1% (w/v) tryptone, 0.5% (w/v) yeast extract, 1% (w/v) NaCl, and 2% (w/v) agar, and incubated at 30°C for 24 h. The size of the clear zone represented the bacterial caseinolytic ability.

#### 2.3.5 Exopolysaccharide production

Culture plates containing 1% (w/v) tryptone, 0.004% (w/v) Congo red, and 0.5% (w/v) agar were used to determine the exopolysaccharide production. Three microliters of overnight cultured bacteria were spotted on the agar plates and incubated at 30°C for 24 h before examination. The higher the production of exopolysaccharides by bacteria, the more intense the red color of the bacterial colony is observed.

#### 2.3.6 Swarming and swimming activities

The plates for the swarming activity assay contain M8 medium and 0.5% (w/v) Bacto agar. For the swimming assay, plates that consist of 1% (w/v) tryptone, 1% (w/v) NaCl, and 0.3% (w/v) agar were prepared (Kohler et al., 2000). Three microliters of overnight cultured bacteria were spotted on the swarming and swimming agar plates and incubated for 24 h at 30°C before examination.

### 2.4 Total RNA extraction and transcriptome analysis

Overnight grown *P. aeruginosa* strains were diluted 100-fold into fresh LB broth supplemented with 500 μg/mL carbenicillin and incubated at 37°C with agitation for 9 h till the late log phase at which point the cell densities of all bacteria were comparable (Supplementary Figure S1). The bacterial RNA was extracted using the TRIzol^®^ reagent (Invitrogen, United States) and further purified using the PureLink^™^ RNA Mini kit (Thermo-Fisher Scientific, United States) as previously described (Lo et al., 2018) according to the manufacturer’s instructions. The contaminating DNA was removed with DNase I (TakaraBio, Japan) along with an RNase inhibitor (GeneDireX, Taiwan). Then the RNA concentration was determined using a NanoDrop ND-1000 spectrophotometer. Complementary DNA was synthesized with GeneScript First-Strand Synthesis Kit with 2 μg RNA and random hexamers following the manufacturer’s instructions. RNA-Seq was performed duplicates using Hiseq 4,000 (Illumina, Inc., San Diego, CA). About 86.5 million and 82.9 million reads were generated for RNA samples from PAO1 [pMMB66EH] and PAO1 [pMMB*psrA*], respectively. The relative gene expression level was determined and normalized based on reads per kilobase per million mapped reads (RPKM).

### 2.5 Quantitative real-time PCR analysis

One microliter of synthesized cDNA was used for qRT-PCR analysis according to the instruction of the Power SYBR Green PCR Master Mix (Applied Biosystems, Warrington, United Kingdom) was used for qRT-PCR analysis. The primers used in the analysis are listed in Supplementary Table S2. The mRNA levels relative to the internal controls (16S rRNA and *proC*) were calculated from triplicate independent experiments using the 2^−ΔΔCt^ approach (Yuan et al., 2006).

### 2.6 Purification of the recombinant PsrA protein

Recombinant PsrA was generated by cloning the *psrA* open reading frame into the expression vector pET30a (Novagen) to obtain plasmid pET30*psrA* and overexpressed in *E. coli* BL21(DE3) under the induction of 1.0 mM isopropyl-ß-D-thiogalactopyranoside (IPTG) for 3 h at 37°C. The cells were harvested by centrifugation at 5,000 *g* for 10 min at 4°C, resuspended in buffer A (20 mM Tris–HCl, pH 7.8, 500 mM NaCl, and 10% glycerol), and disrupted by sonication. Subsequently, the supernatant containing soluble protein lysate obtained from centrifugation at 14,000 *g* for 30 min at 4°C was filtered through a 0.45 μm filter to avoid any remaining cell debris and loaded onto a Ni-charged column. Purification of the C-terminal hexahistidine-tagged PsrA was conducted under conditions recommended by the manufacturer (Thermo-Fisher Scientific, United States). The protein was eluted from the column with buffer A containing 300 mM imidazole and dialyzed against buffer containing 20 mM Tris–HCl, pH 7.8, 200 mM NaCl, and 20% glycerol at 4°C overnight. The concentration of PsrA was determined by the Bradford assay.

### 2.7 Electrophoretic mobility shift assay

A modified electrophoretic mobility assay (EMSA) was performed as described previously (Kang et al., 2009), approximately one microgram of PsrA was incubated with 100 ng of testing DNA fragment in a binding buffer containing 25 mM 4-(2-hydroxyethyl)-1-piperazine ethanesulfonic acid, pH 7.6, 15 mM NaCl, 1 mM dithiothreitol, 20 mM bovine serum albumin, 0.1 mM EDTA at room temperature for 30 min. The PsrA-DNA mixture was resolved on a 6% polyacrylamide gel in Tris-borate-EDTA buffer under a constant voltage of 120 V for 90 min at 4°C. The gel was stained with SYBR^™^Safe DNA gel stain (Invitrogen, United States) and examined under UV illumination.

### 2.8 Western blot analysis

A 723-bp fragment of DNA sequence containing the *psrA* coding sequence and a FLAG (DYKDDDK) tag was generated by PCR using *P. aeruginosa* PAO1 as the template and cloned into the plasmid pMMB66EH. The resulting construct was conjugated to *P. aeruginosa* PAO1 and grown overnight with or without IPTG induction. The cells were harvested by centrifugation, and aliquots of protein lysate were resolved by electrophoresis on a 4–20% SDS-polyacrylamide gel and electrophoretically transferred to a nitrocellulose membrane (GE HealthCare, Chicago, United States). After incubation with 5% skim milk to block non-specific binding of antibodies, the membrane underwent a two-step immunoreaction. First, it was incubated with rabbit polyclonal anti-FLAG antibody (Elabscience, Texas, United States) diluted 1:5,000 in TBST (20 mM Tris–HCl, pH 7.6, 150 mM NaCl, and 0.2%(v/v) Tween-20) followed by horseradish peroxidase-conjugated goat anti-rabbit antibody (GeneTex, Taiwan) diluted 1:5,000 in TBST. The presence of PsrA-FLAG was detected using the chemiluminescence method with ECL substrate (GeneTex, Taiwan) and visualized with a biomolecular imager ImageQuant LAS4000 Mini (GE HealthCare, United States).

### 2.9 N-acyl homoserine lactone assays

The short-chain autoinducer C_4_-HSL bioassay was conducted using *Chromobacterium violaceum* CV026 as a reporter (McClean et al., 1997). The assay plates consisted of a 10 mL bottom layer of 1.5% LB agar and a 10 mL top layer of 0.3% LB agar containing 200 μL of overnight grown *C. violaceum* CV026. A well 7.5 mm in diameter was made at the center of the upper agar layer, and 3 μL of overnight-grown *P. aeruginosa* were added to the well. The plate was then incubated at 30°C and the production of the purple pigment violacein around the wells in response to C_4_-HSL was examined at 24 h.

The detection of long-chain autoinducer 3-oxo-C_12_-HSL was performed using *E. coli* JM109 [pSB1075] as a reporter (Winson et al., 1998). Briefly, the testing *P. aeruginosa* culture filtrate was collected by centrifugation at 5,000 g for 10 min, and the supernatant was filtered through a 0.45 μm filter. In a 96-well microtiter plate, 100 μL of 10× LB-diluted JM109 [pSB1075] was mixed with 100 μL 10× LB-diluted culture filtrate, and the plate was incubated at 30°C for 4 h with rocking at 150 rpm. The bioluminescence signal was measured using a fluorescence multilabel reader (Wallac VICTOR3) and calculated as a relative light unit per optical density at 595 nm of the culture (RLU/OD595).

### 2.10 Statistical methods

All data were presented as mean ± standard error of the mean for each group. Statistical analyses were performed using student’s *t*-test, one-way analysis of variance (ANOVA), or two-way ANOVA with Tukey’s multiple comparison test. *p* values of <0.05 were considered statistically significant. The statistical significance was set to *p < 0.05, **p < 0.01, ***p < 0.001, and ****p < 0.0001. All error bars represent the standard error of the mean. The analyses were performed using Prism 6 (GraphPad software).

## 3 Results

### 3.1 PsrA influenced quorum sensing in *P. aeruginosa*

**Figure 1:**
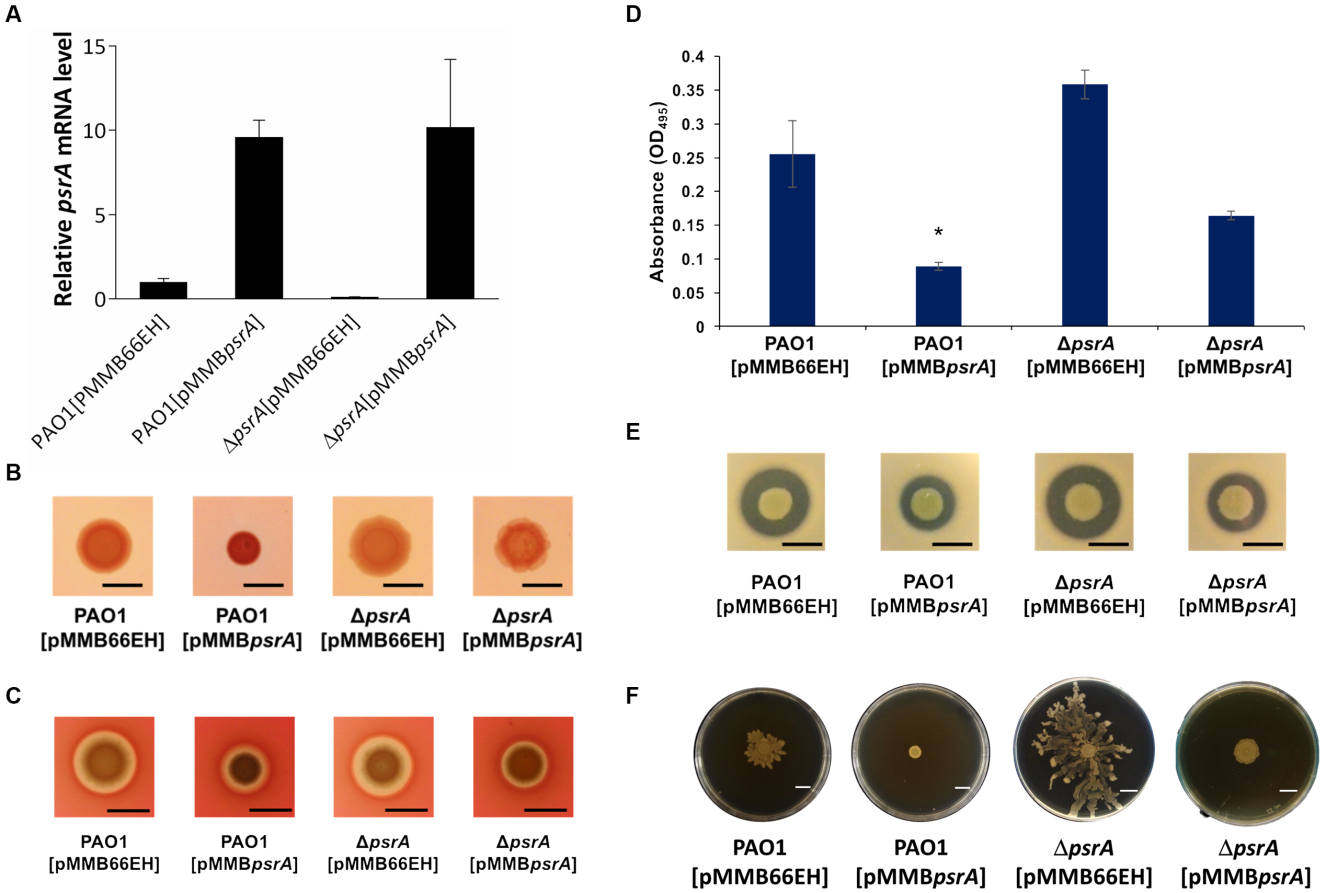
Effects of PsrA on gene expression of *psrA*, exopolysaccharide production, hemolysis, elastase, caseinase activity and swarming activity in *P. aeruginosa* PAO1. **(A)** Real-time quantitative PCR analysis of the effect of PsrA on *psrA* gene expression. The relative quantity of *psrA* transcript levels was compared with that of PAO1. The data represents means of triplicates ± standard errors. **(B)** Exopolysaccharide production. Three microliters overnight bacterial culture of each strain was inoculated on Congo red agar and incubated at 37°C for 24 h. Scale bar = 1 cm. **(C)** Hemolysis activity. Three microliters overnight bacterial culture of each strain was inoculated on blood agar plates, and the hemolytic zone was examined after incubation of the plates at 37°C for 24 h. Scale bar = 1 cm. **(D)** Elastase activity. The supernatant of the overnight bacterial culture was incubated with a solution containing elastin-Congo red with shaking at 37°C for 4 h. The release of Congo red by elastase was measured at OD_495_. The data represent means of quadruplicates ± standard errors. *p < 0.05. **(E)** Caseinase activity. Three microliters overnight bacterial culture of each strain was inoculated on casein agar plates, and the casein hydrolytic activity was examined after incubation of the plates at 37°C for 24 h. Scale bar = 1 cm. **(F)** Swarming activity. M8 medium containing 0.5% agar was used in the swarming motility assay. Three microliters overnight bacterial culture of each strain was inoculated on swarming plates and observed after incubation at 30°C for 24 h. Scar bar = 1 cm.

To bypass the self-repressing nature of PsrA, we cloned the *psrA* gene into pMMB66EH, replacing its native promoter with an IPTG-inducible promoter. Quantitative RT-PCR analysis of the *P. aeruginosa* harboring the plasmid pMMB*psrA* confirmed that this resulted in significantly higher *psrA* expression in the *P. aeruginosa* strain harboring pMMB*psrA* compared to wild-type PAO1 (Figure 1A). A Western blot analysis was also performed to confirm that PsrA was produced in a higher quantity in PAO1 [pMMB*psrA*] than its parental counterpart (Supplementary Figure S2). To evaluate the effect of the increased expression of *psrA* on *P. aeruginosa* PAO1, we conducted RNA-Seq analyses comparing the transcriptomes of PAO1 [pMMB66EH] and PAO1 [pMMB*psrA*]. Approximately 550 genes were upregulated, and 1,200 genes were downregulated more than twofold in PAO1 [pMMB*psrA*] compared to PAO1 [pMMB66EH] (Supplementary Table S3). Notably, the expression of several quorum sensing-related genes was altered in PAO1 [pMMB*psrA*]. Examples of *psrA*-upregulated genes included the PQS quorum sensing operon (*pqsABCDE/phnAB*) and most genes of the pyocyanin synthesis operons (*phz1* and *phz2*). Conversely, major virulence genes with reduced expression in PAO1 [pMMB*psrA*] included those encoding siderophore synthesis (*fepCBDG*), pyoverdine synthesis (PA2384-PA2415), the type III secretion system (T3SS; PA1694-PA1721), alginate synthesis (PA3544-PA3551), lipid A modification enzymes (PA3552-PA3559), the major elastase LasB, and the caseinase Piv. The detailed sequencing data of RNA-Seq has been deposited to Gene Expression Omnibus (GEO) under the accession number GSE249518.

**Figure 2:**
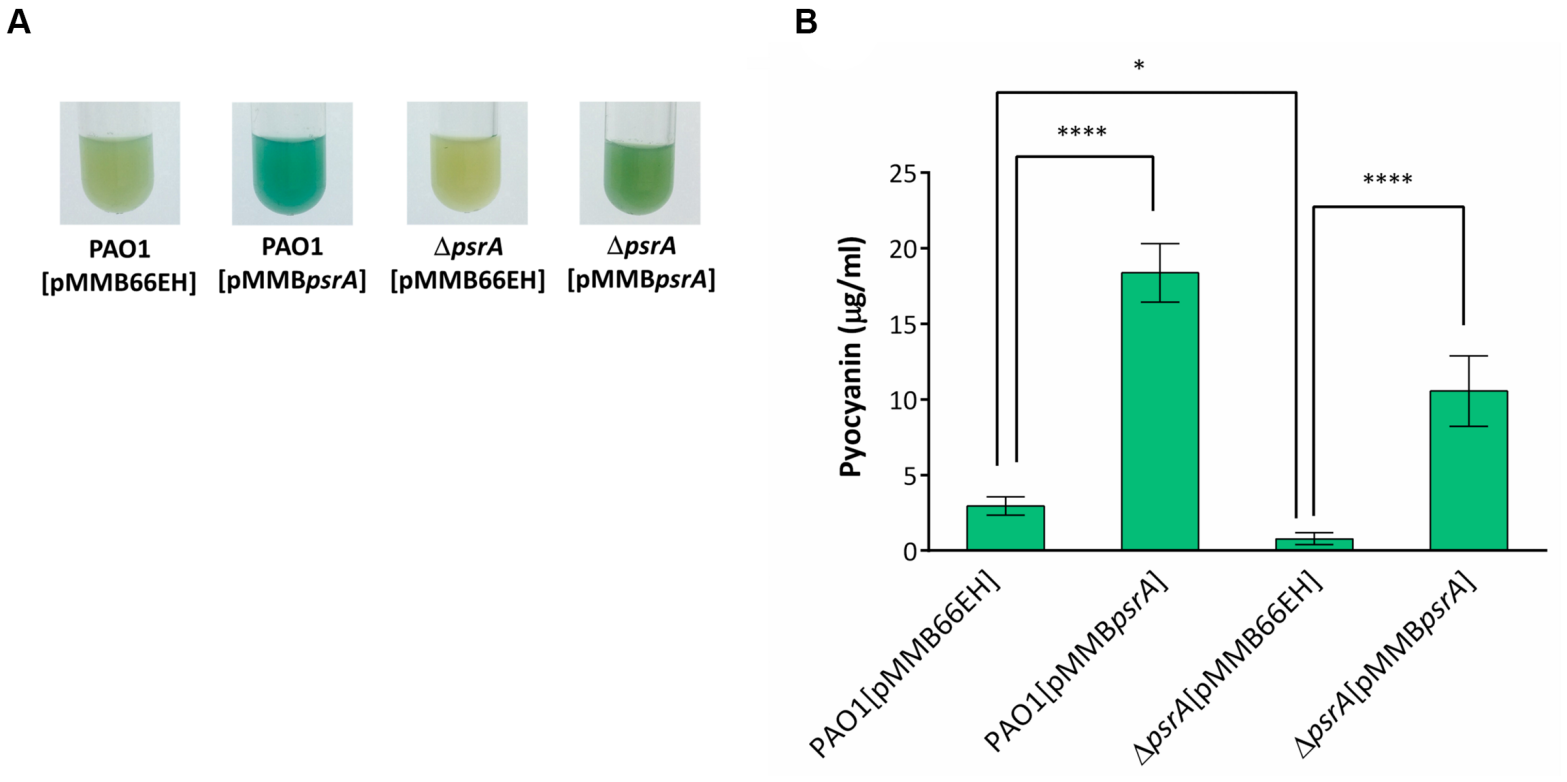
Effects of PsrA on pyocyanin production of *P. aeruginosa* PAO1. **(A)** Pyocyanin production, reflected by the intensity of the blue-greenish color, of different *P. aeruginosa* recombinant strains after growth in LB broth for 18 h. **(B)** Spectrophotometric analysis of pyocyanin produced by different *P. aeruginosa* PAO1 recombinant strains. The values represent mean ± S.D. from three biological repeats. Multiple comparisons between groups were assessed by one-way ANOVA followed by Tukey’s comparison test (*p < 0.05 and ****p < 0.001).

We validated the transcriptomic findings by examining several quorum sensing-regulated phenotypes. In line with the gene expression data, pyocyanin synthesis significantly increased in PAO1 [pMMB*psrA*] compared to the wild-type strain up to ~6 folds (Figure 2). Meanwhile, the ∆*psrA* mutant displayed decreased pyocyanin production. These findings are aligned with an earlier study reporting that deletion of *psrA* decreased the production of PQS and pyocyanin (Wells et al., 2017). Introducing pMMB*psrA* into the ∆*psrA* strain also significantly enhanced pyocyanin production (Figure 2). Contrasting patterns emerged for other phenotypes: exopolysaccharide, elastase, hemolysis, caseinase, and swarming activities all decreased in PAO1 [pMMB*psrA*] but showed subtle increases in the *psrA* knockout strain (Figures 1B–F).

### 3.2 PsrA affected *lasR* and *rhlR* expression in *P. aeruginosa*

**Figure 3:**
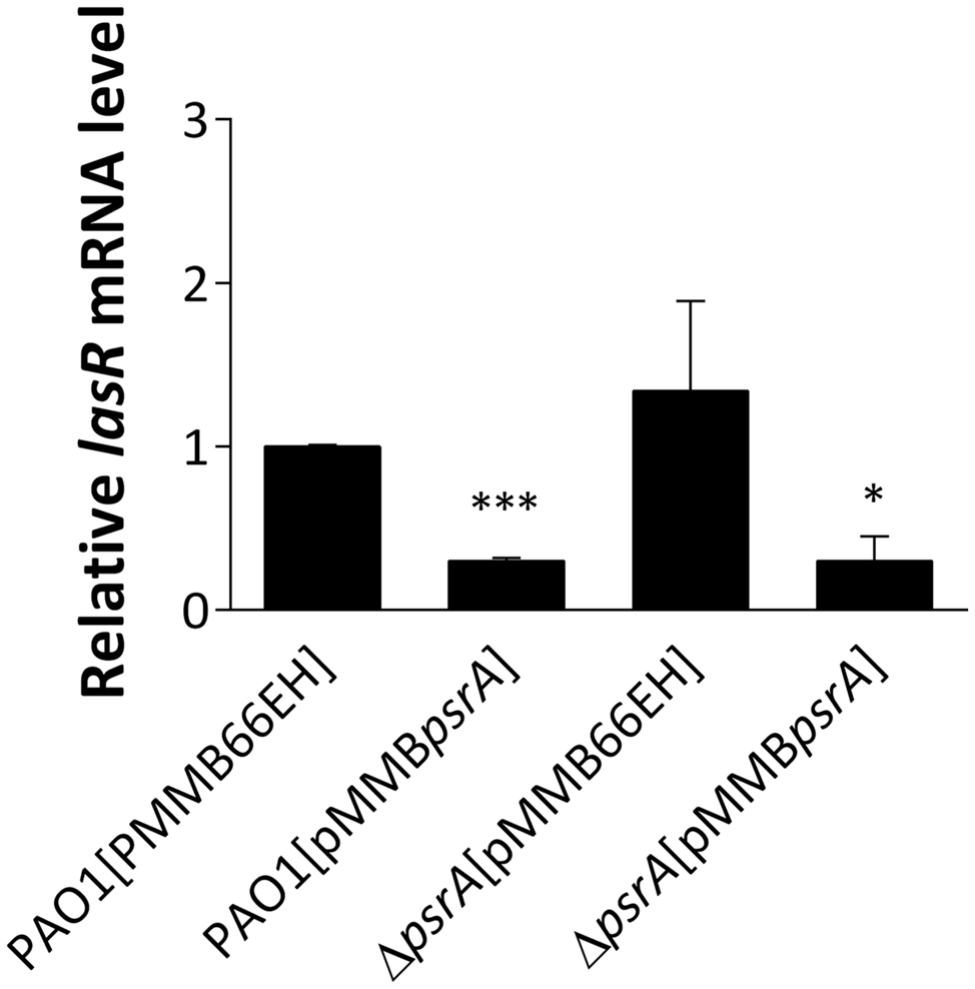
Real-time quantitative PCR analysis of the effect of PsrA on *lasR* gene expression. The relative quantity of *lasR* transcript levels was compared with that of PAO1. The data represent means ± standard errors from three independent experiments, *p*-value was supported by Student’s *t*-test (*p < 0.05 and ***p < 0.001).

Interestingly, downregulated expression of *lasR* and *rhlR*, two master regulators of quorum sensing in *P. aeruginosa* PAO1 [pMMB*psrA*], was noted (Supplementary Table S3), though the decrease of *lasR* is more significant than *rhlR* (9.3- and 2.5-fold respectively). The decreased expression of *lasR* was again validated using qRT-PCR confirming that the *lasR* RNA in PAO1 [pMMB*psrA*] was reduced to approximately one-fourth of that in PAO1 [pMMB66EH] (Figure 3). The repression of these key signaling systems indicates that PsrA acts as a negative regulator of *lasR*, regulating cell-to-cell communication in *P. aeruginosa* PAO1.

### 3.3 PsrA affected the production of acyl-homoserine lactone autoinducers

**Figure 4:**
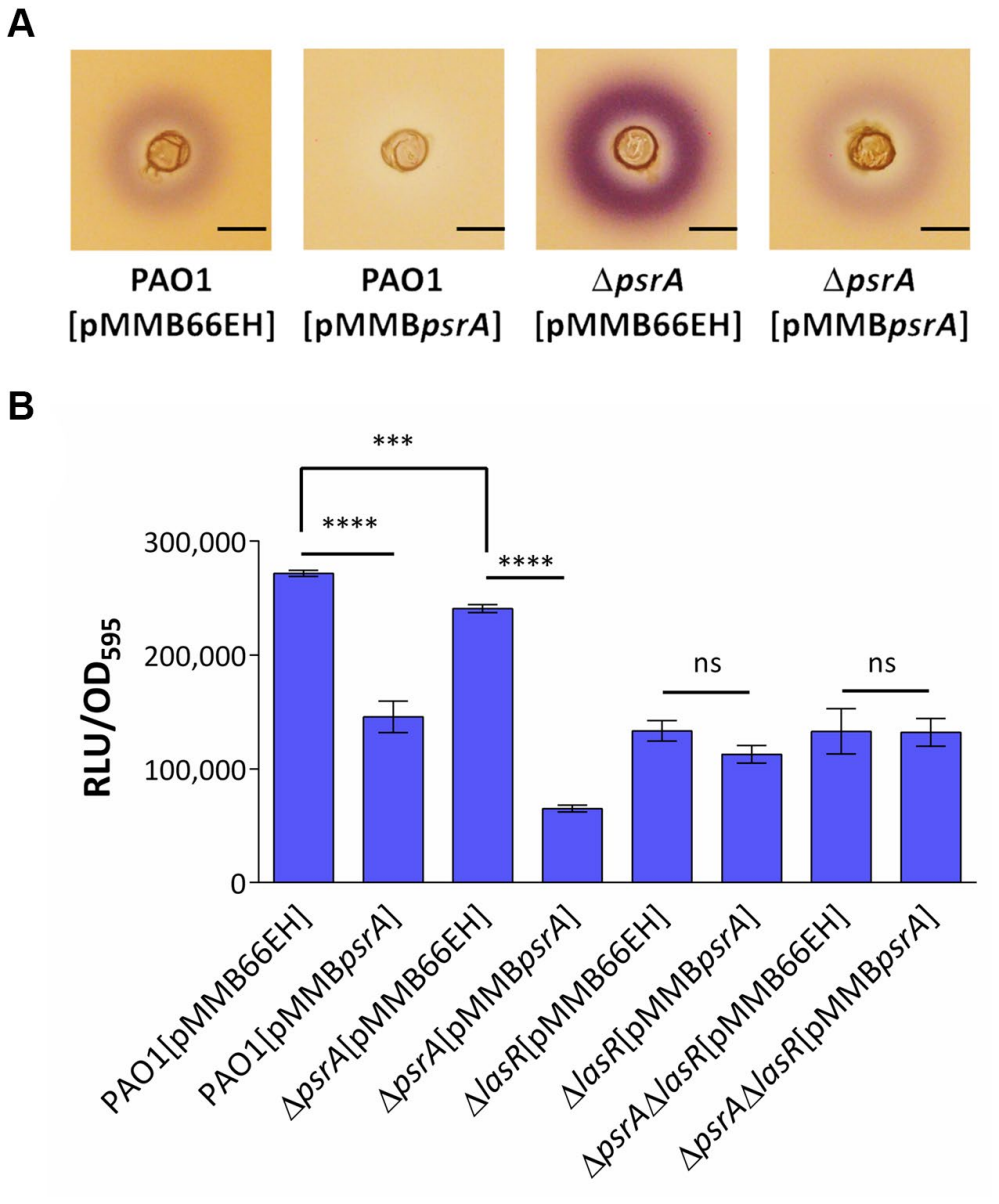
Assessment of PsrA effects on the production of AHL signals. **(A)** *Chromabacterium violaceum* CV026 plate assay. The plate was prepared by overlaying a top LB layer containing *C. violaceum* CV026 on a pre-solidifying LB agar layer. A well was made in the middle of the top layer. Then three microliters of overnight bacterial culture were added to wells. The purple violacein zone surrounding the well generated by *C. violaceum* CV026 in response to the presence of C_4_- to C_8_-HSL was examined. Scale bar = 1 cm. **(B)** *Escherichia coli* JM109 [pSB1075] biosensor assay. Overnight bacterial culture supernatant was co-cultured with *E. coli* harboring *N*-acyl homoserine lactone sensor plasmids, pSB1075 for 4 h. Bioluminescent signals in relative light units (RLU) conferred by pSB1075 in the presence of 3-oxo-C_12_-HSL and the culture optical density at 590 nm (OD595) were recorded. All error bars represent the standard error of the mean. Multiple comparisons were evaluated by one-way ANOVA with Tukey’s test. ***p < 0.001 and ****p < 0.001; ns, not significant, *n* = 5.

To further verify whether PsrA regulates quorum sensing in *P. aeruginosa* and given that the Rhl system produces C_4_-HSL while the Las system produces 3-oxo-C_12_-AHL, we utilized two acyl homoserine lactone (AHL) reporters to specifically determine each AHL type. *Chromobacterium violaceum* CV026 produces a purple pigment called violacein in a dose-dependent manner when exposed to C_4_- to C_8_-HSL, making it a suitable reporter for detecting C_4_-HSL produced by *P. aeruginosa* PAO1 (McClean et al., 1997). As shown in Figure 4A, the strain PAO1 [pMMB*psrA*] showed significantly diminished production of C_4_- to C_8_-HSL compared to control strains, as evidenced by less purple pigmentation from *C. violaceum* CV026. Conversely, the deletion of *psrA* triggered augmented violacein production from *C. violaceum* CV026, further indicating the negative effect of PsrA on C_4_-HSL production in *P. aeruginosa* PAO1. This finding aligns with RNA-Seq data revealing that PsrA overexpression represses *rhlR*, a key regulator that governs short-chain AHL synthesis. Additionally, culture supernatants were tested using the *E. coli* JM109 [pSB1075] bioluminescent sensor specific for the 3-oxo-C_12_-HSL signal (Winson et al., 1998). Consistent with the transcriptomic results indicating *lasR* repression in PAO1 [pMMB*psrA*], the higher expression of PsrA also reduced 3-oxo-C_12_-HSL levels, which is normally regulated by LasR. Together, these findings substantiate that PsrA perturbs multiple quorum sensing circuit nodes, congruent with its transcriptionally regulating cognate signaling genes (Figure 4B).

### 3.4 The effects of PsrA on AHL production were likely mediated through LasR

Given LasR’s integral role governing quorum sensing, we examined if LasR mediates the autoinducer reductions by PsrA. Using the 3-oxo-C_12_-HSL bioreporter again, *lasR* deletion in PAO1 expectedly decreased 3-oxo-C_12_-HSL levels. The strain PAO1 [pMMB*psrA*] also showed a similar lowering. However, the increased *psrA* expression levels in a ∆*lasR* background did not further diminish 3-oxo-C_12_-HSL (Figure 4B), indicating the absence of additive effects. This lack of synergy suggests the impact of PsrA on 3-oxo-C_12_-HSL occurs through LasR. Overall, the data fit a model where PsrA may act through LasR repression to achieve downstream quorum sensing phenotypes.

### 3.5 LasR also mediated several PsrA phenotypes

**Figure 5:**
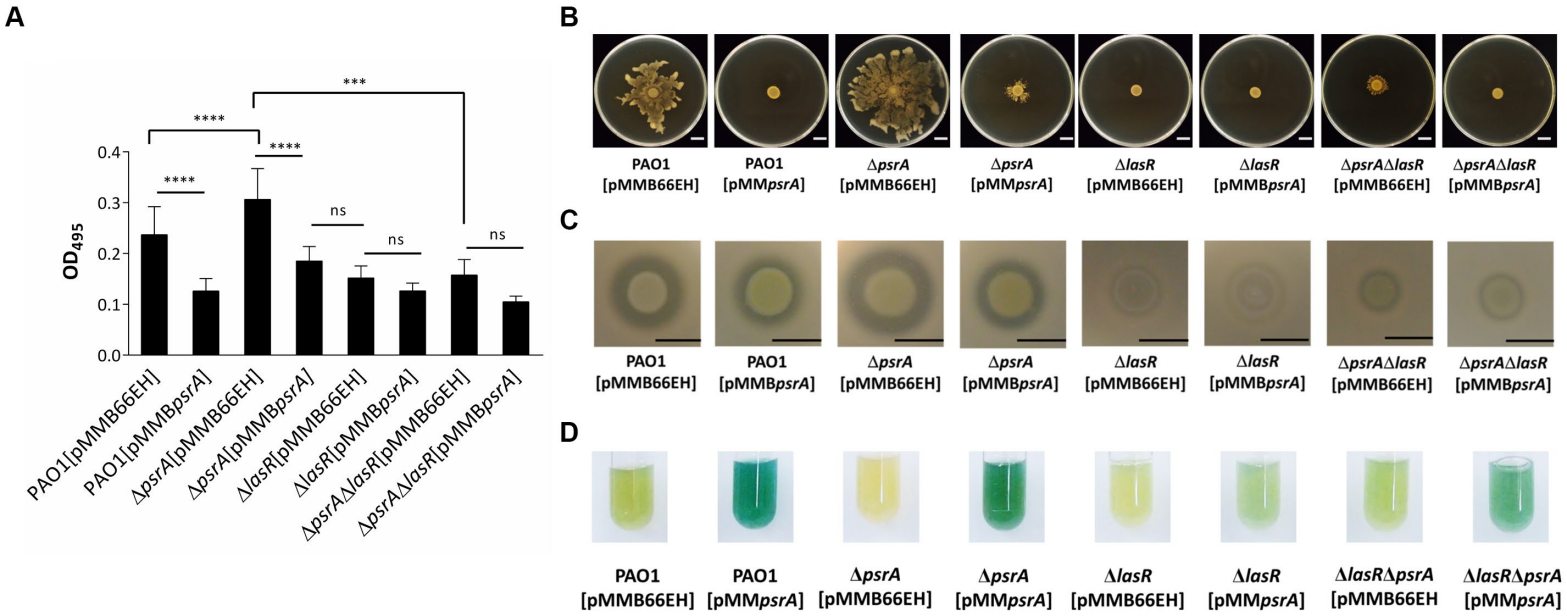
Effects of PsrA in *P. aeruginosa* PAO1 *lasR* deletion on quorum-sensing related phenotypes. **(A)** Elastase activity. The supernatant from the overnight bacterial culture was incubated with an elastin-Congo red solution, shaking at 37°C for 4 h. The release of Congo red by elastase was measured at OD_495_. The data represent ± standard errors from independent quadruplicates. Multiple comparisons were evaluated by one-way ANOVA with Tukey’s test. ***p < 0.001 and ****p < 0.0001; ns, not significant. **(B)** Swarming activity. Three microliters overnight bacterial culture of each strain was inoculated on M8 medium plates containing 0.5% agar and observed after incubation at 30°C for 24 h. Scar bar = 1 cm. **(C)** Caseinase activity. Three microliters overnight bacterial culture of each strain was inoculated on casein agar plates and examined after incubation at 37°C for 24 h. Scale bar = 1 cm. **(D)** Pyocyanin production. Pyocyanin production of each bacterial strain was examined after 18 h incubation at 37°C.

We next investigated if PsrA influences additional LasR-regulated phenotypes like virulence factor production. Both PAO1 [pMMB*psrA*] and the Δ*lasR* [pMMB66EH] showed reduced elastase activity (Figure 5A). However, introducing pMMB*psrA* into the Δ*lasR* mutant did not enhance the decreased levels of elastase, indicating that PsrA requires LasR to manifest repression. Moreover, the Δ*lasR*Δ*psrA* double knockout displayed similar elastase levels as the Δ*lasR* strain. This LasR-dependent pattern held for other quorum sensing related phenotypes including swarming motility and caseinase activity (Figures 5B,C). A subtly different trend emerged for pyocyanin synthesis; the *psrA* overexpression Δ*lasR* strain retained some pyocyanin not seen in the single Δ*lasR* mutant (Figure 5D). This suggests that while PsrA predominantly utilizes LasR to modulate virulence outputs, additional regulatory pathways are likely to contribute to pyocyanin production.

### 3.6 PsrA binds directly to the *lasR* promoter

**Figure 6:**
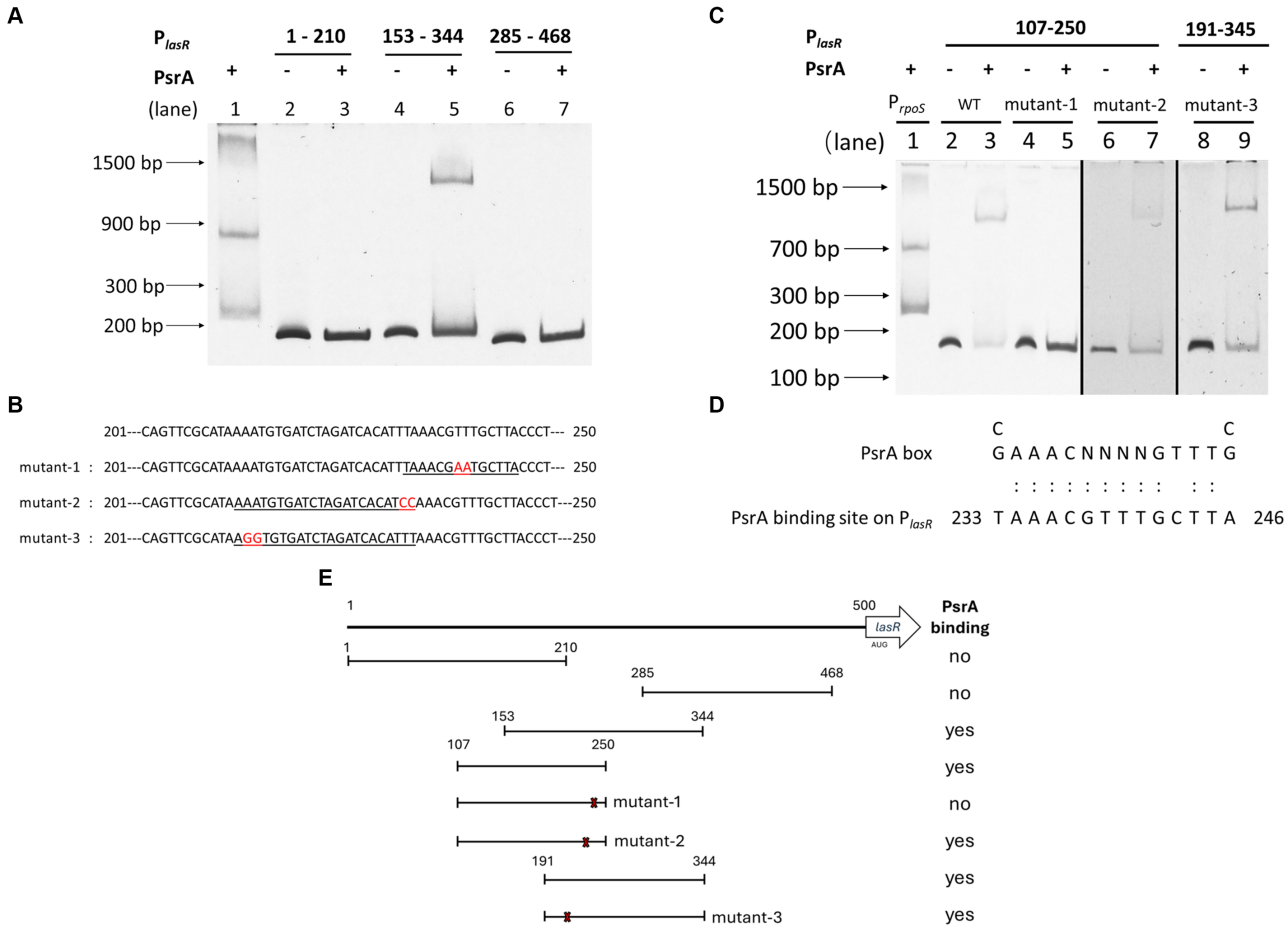
Identification of PsrA binding site on the *lasR* promoter using electrophoretic mobility shift assay (EMSA). **(A)** Localization of PsrA binding site to a 200 bp region. The DNA fragments used in the study areas are as follows. Lanes 1, *P*_rpoS_ as a positive control; 2, *P*_lasR_ 1–210 bp; 3, *P*_lasR_ 1–210 bp with PsrA protein; 4, *P*_lasR_ 153–344 bp; 5, *P*_lasR_ 153–344 bp with PsrA protein; 6, *P*_lasR_ 285–468 bp; 7, *P*_lasR_ 285–468 bp with PsrA protein. **(B)** The nucleotide sequences of the two-nucleotide substitutions in mutant-1, mutant-2, and mutant-3 for identification of the PsrA binding sequence are marked in red. Underlined is the predicted PsrA binding sequence. **(C)** Identification of the specific binding site of PsrA on *P*_lasR._ DNA fragments used in each lane are 1, positive control *P*_rpoS_; 2, *P*_lasR_ 107–250 bp; 3, *P*_lasR_ 107–250 bp with PsrA; 4, *P*_lasR_ 107–250 bp (mutant-1); 5, *P*_lasR_ 107–250 bp (mutant-1) with PsrA; 6, *P*_lasR_ 107–250 bp (mutant-2); 7, *P*_lasR_ 107–250 bp (mutant-2) with PsrA; 8, *P*_lasR_ 191–344 bp (mutant-3); 9, *P*_lasR_ 191–344 bp (mutant-3) with PsrA. Negative images of the SYBR Safe-stained gel are shown. **(D)** Comparison of PsrA binding sequence on *lasR* promoter and the known PsrA box. The numbers indicate the nucleotide position of the PsrA binding site on the *lasR* promoter. **(E)** Schematic diagram showing the relative positions of different DNA fragments comprising different regions of the *lasR* promoter. The numbers represent the nucleotide positions of each testing truncated *lasR* promoter region. The locations of three site-specific nucleotide substitutions (mutant-1, mutant-2, and mutant-3) are marked with an × sign. A summary of recombinant PsrA protein binding is shown on the right.

To determine whether PsrA directly controls *lasR* gene expression, an affinity-purified PsrA was tested for its binding to several DNA fragments comprising the putative *lasR* promoter (Supplementary Figure S3) in an EMSA. Figure 6A demonstrates that PsrA is capable of binding to the *lasR* promoter at nucleotide position 153–285 bp. Using serial truncated DNA fragments of the *lasR* promoter has allowed us to further locate the PsrA binding site to a 50 bp region *lasR* promoter at nucleotide positions 201–250. Three DNA fragments (mutant-1, mutant-2, and mutant-3) with different nucleotide substitutions were generated based on two predicted PsrA binding sites within the 50 bp region (Figure 6B). Further analysis of these synthetic DNA fragments revealed that the sequence TTAAACGTTTGCTTAC is essential for the binding of PsrA (Figure 6C). The sequence exhibited moderate (Figure 6D) homology with the PsrA binding motif G/CAAACN_(2–4)_GTTTG/C reported previously (Kojic et al., 2002). A summary of the DNA fragments selected for EMSA and the binding result is depicted in Figure 6E. Attention is drawn to the question of whether PsrA might also regulate another pivotal quorum-sensing regulator, such as RhlR or the PQS regulator PqsR (MvfR), both of which play a role in pyocyanin production. However, the negative binding results between PsrA and the promoter regions of these two genes from the EMSAs (Supplementary Figure S4) suggest that PsrA may not directly regulate these regulators. Indeed, the downregulation of the quorum-sensing-related genes appears to be a result of reduced *lasR* expression.

### 3.7 Oleate affects PsrA binding to the *lasR* promoter

**Figure 7:**
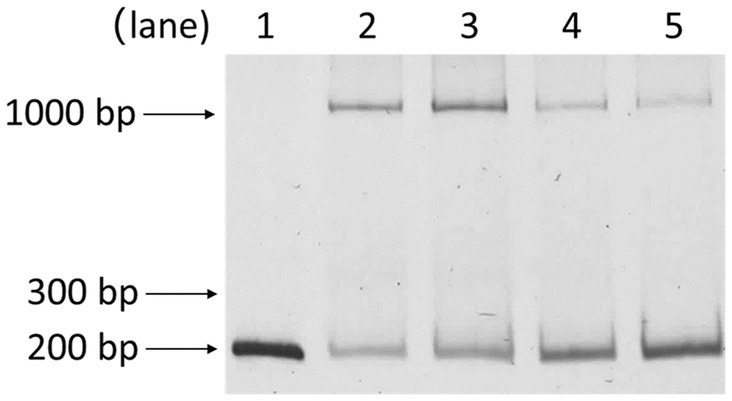
Effect of oleate on the binding of PsrA with *P*_lasR_ 153–344 bp. Lanes 1, *P*_lasR_ 153–344 bp only; 2, *P*_lasR_ 153–344 bp with PsrA; 3, *P*_lasR_ 153–344 bp with PsrA and 30 μM oleate; 4, *P*_lasR_ 153–344 bp with PsrA and 60 μM oleate; 5, *P*_lasR_ 153–344 bp with PsrA and 100 μM oleate. Negative images of the SYBR Safe-stained gel are shown.

Building upon prior works showing PsrA responding to long-chain fatty acids in regulating the *fadBA5* beta-oxidation operon, this study explored the potential influence of oleate on PsrA binding to the *lasR* promoter. As demonstrated in Figure 7, oleate effectively blocked PsrA binding at a concentration of 30 μM, paralleling the concentration known to inhibit PsrA binding to the *fad* promoter (Kang et al., 2008, 2009).

### 3.8 Oleate affects PsrA related quorum sensing phenotypes

**Figure 8:**
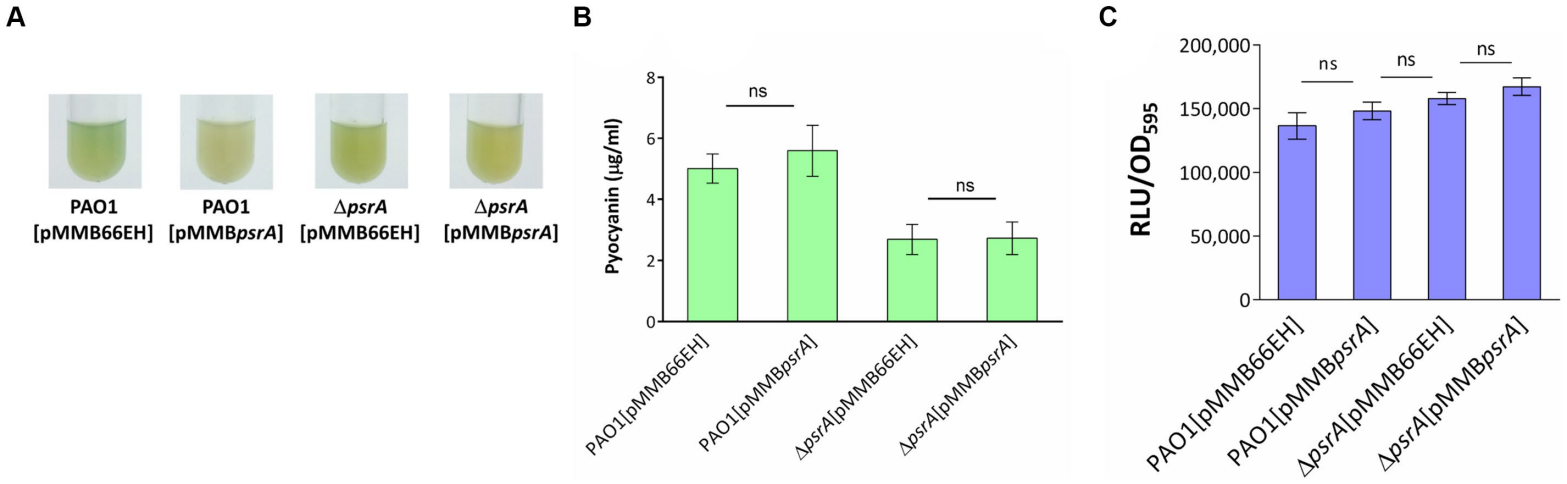
Effect of oleate on the PsrA-mediated pyocyanin and 3-oxo-C_12_-AHL production. **(A)** *P. aeruginosa* PAO1 strains were tested for their pyocyanin production in the presence of 1 mM oleate for 18 h at 37°C. **(B)** Spectrophotometric analysis of pyocyanin produced by different *P. aeruginosa* PAO1 recombinant strains under the effect of 1 mM oleate. The values represent mean ± S.D. from three biological repeats. Multiple comparisons between groups were assessed by one-way ANOVA followed by Tukey’s comparison test (ns, not significant). **(C)** The 3-oxo-C_12_-AHL released by the bacteria in response to oleate was evaluated with a bioluminescence-based JM109 [pSB1075] reporter system. The data represent the values of means independent triplicates ± standard errors. Multiple comparisons were evaluated by one-way ANOVA with Tukey’s test. ns, not significant.

The *psrA* overexpressing strain exhibited a hyper pyocyanin production phenotype (Figure 2). The addition of 1 mM oleate in the culture maintained the pyocyanin synthesis of PAO1 [pMMB*psrA*] and Δ*psrA* [pMMB*psrA*] at the wild-type level suggesting that the effect of PsrA was disrupted by oleate (Figures 8A,B). Likewise, the addition of oleate alleviated the inhibition effect of PsrA on 3-oxo-C_12_-HSL production in *psrA* overexpressing strain of PAO1 and Δ*psrA*, emphasizing the tuning role of oleate in the PsrA-mediated regulation in *P. aeruginosa* PAO1 (Figure 8C). Taken together, these findings suggest a common mode of PsrA regulation by fatty acids across distinct operons.

## 4 Discussion

**Figure 9:**
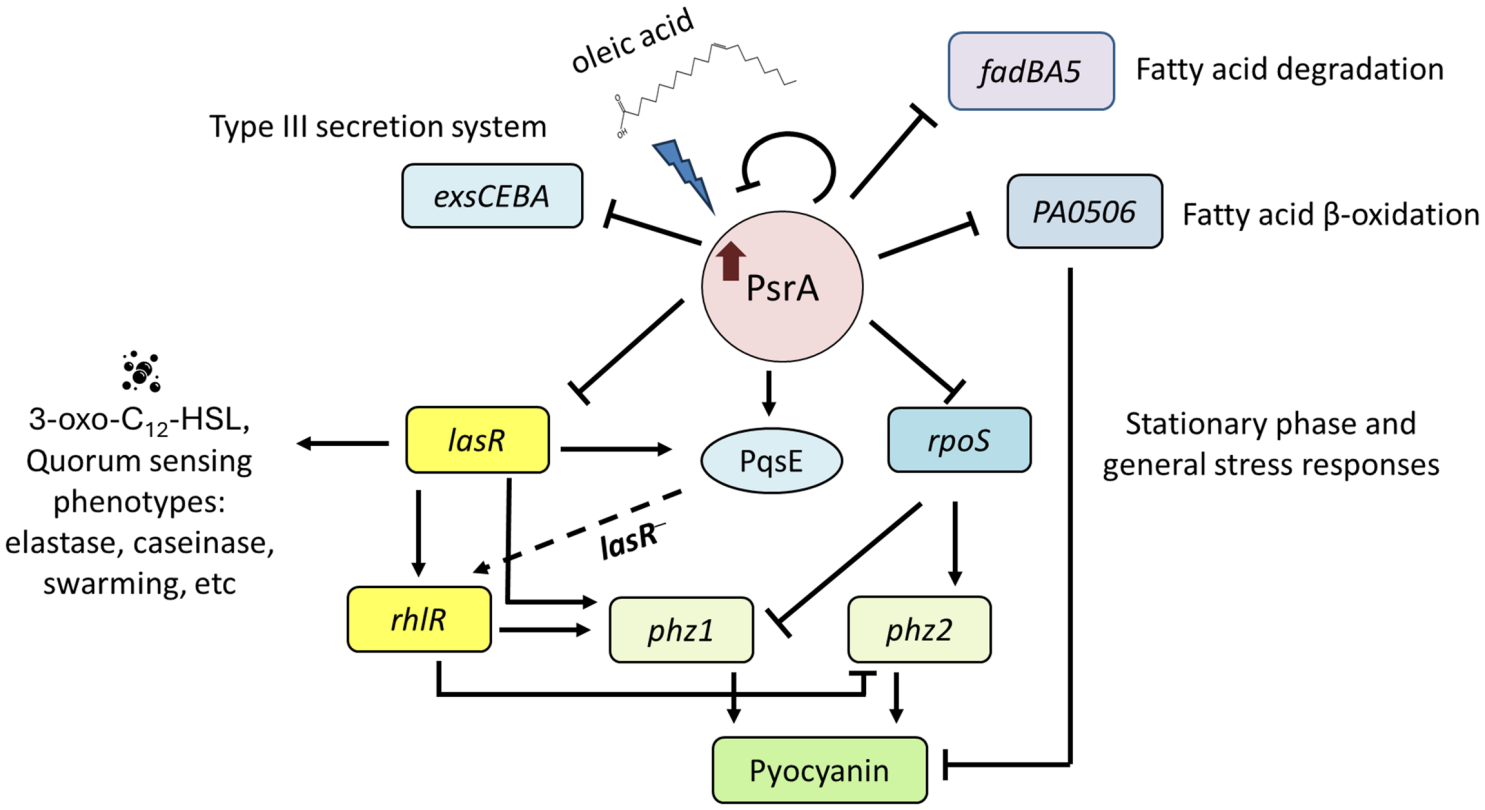
Proposed function model of PsrA regulon. PsrA serves as a regulator of quorum sensing through direct regulation of *lasR* at the transcription level. It negatively regulates 3-oxo-C_12_-HSL-based quorum-sensing regulator *lasR* and promotes LasR downstream virulence-related activities, such as pyocyanin caseinase, elastase, hemolysis, swarming activities, and RhlR-regulated genes. Meanwhile, oleic acid reverses the repression effect of PsrA. Moreover, PsrA has been reported to regulate the expression of genes encoding the general stress sigma factor RpoS, fatty acid degradation regulator FadBA5, acyl-coA dehydrogenase PA0506, and type III secretion systems. The dashed line indicates a possible regulatory role of PqsE to *rhlR* in the absence of LasR.

This study demonstrated that PsrA overexpression induced pyocyanin production (Figure 2) but suppresses multiple quorum-sensing-related phenotypes, including exopolysaccharide production, hemolysis activity, elastase activity, caseinase activity, and motility (Figure 1). In addition, this study demonstrated that PsrA repressed quorum sensing-associated phenotypes through direct regulation of *lasR* at the transcription level (Figures 3, 6). Consistent with the notion, the increased expression of *psrA* in a Δ*lasR* background did not further reduce 3-oxo-C_12_-HSL levels, suggesting that PsrA’s impact on 3-oxo-C_12_-HSL production operates through LasR (Figure 4). Notably, the Δ*lasR*Δ*psrA* double mutant displayed similar levels of elastase, caseinase, and swarming activities observed in the Δ*lasR* strain, reinforcing this dependence (Figure 5). Although PsrA disturbed the production of C_4_-HSL (Figure 4), there is no evidence from this study to show that the repression effects on RhlR and PqsR are direct (Supplementary Figure S4). Thus, together with AlgR2, GacA/GacS, MvaT, RpoN, and Vfr (Albus et al., 1997; Parkins et al., 2001; Diggle et al., 2003; Heurlier et al., 2003; Ledgham et al., 2003; Thompson et al., 2003), PsrA can be regarded as another regulator of quorum sensing in *P. aeruginosa* (Figure 9).

In addition to acting as a regulator of quorum sensing, PsrA has been reported to directly regulate the expression of the general stress regulator RpoS (Kojic and Venturi, 2001; Chatterjee et al., 2007; Kang et al., 2009; Wells et al., 2017). RpoS positively regulates the expression of approximately 500 genes and suppresses 270 genes in the stationary phase of *P. aeruginosa* (Schuster et al., 2004). How PsrA coordinately regulates the expression of RpoS and LasR regulons is intricate. For example, it has been reported that the expression of *rpoS* is abolished in a *P. aeruginosa lasR* mutant (Latifi et al., 1996). Another study revealed that the deletion of *rpoS* increased the expression of *lasR* (He et al., 2019). The coordination of RpoS and LasR regulons is critical for the bacterium to survive in hostile environments, and how this is achieved is not fully understood. This study highlights the significance of PsrA in the regulation of these two important regulons, although the detailed mechanism and the spectrum of the signal molecules sensed by PsrA regulation remain to be identified.

LasR has been reported as a positive regulator of pyocyanin production (Gallagher et al., 2002; Schuster and Greenberg, 2006). However, in our study, substantial amounts of pyocyanin were still detected in the PsrA-overexpressing strain despite the absence of LasR (Figure 5D), indicating that LasR is not the sole mediator of PsrA-mediated pyocyanin overproduction. Identifying additional factors involved in PsrA-enhanced pyocyanin production is challenging, partly due to the presence of two independently regulated pyocyanin biosynthesis systems, Phz1 and Phz2, in *P. aeruginosa*. One possible player is FadE, a key enzyme in the fatty acid β-oxidation pathway. Since PsrA is a known repressor of *fadE* (*PA0506*) expression, its overexpression could lead to the accumulation of acyl-CoA, which in turn promotes the synthesis of PQS and pyocyanin (Wells et al., 2017). Another potential factor is the general stress sigma factor RpoS, which downregulates *phz1* expression while upregulating *phz2* expression (Sun et al., 2019). An *rpoS* deletion mutant exhibited enhanced pyocyanin production (Suh et al., 1999), suggesting that PsrA-mediated *rpoS* downregulation may contribute to increased pyocyanin production. Additionally, in the absence of LasR, PqsE’s role in Rhl-dependent quorum-sensing activation becomes prominent (He et al., 2019; Groleau et al., 2020). PqsE, a thioesterase dispensable for PQS biosynthesis, was induced by PsrA (Supplementary Table S3), potentially enabling the bacteria to produce high levels of pyocyanin without LasR. Whether PsrA-induced repression of *lasR* will lead to the development of a LasR-independent Rhl quorum-sensing system remains to be elucidated.

Although PsrA plays a crucial role in *P. aeruginosa* physiology, information regarding its effectors remains limited. This transcription factor is known to respond to long-chain fatty acid signals to regulate the *fadBA5* β-oxidation operon (Kang et al., 2008). However, the specificity and relative potency of different fatty acids on the promoter-binding activity of PsrA have not been fully characterized. This is particularly intriguing because certain fatty acids serve as versatile communication signals within and between bacterial species (Cortes-López et al., 2020). For example, branched-chain fatty acids and myristic acid (C14:0) influence motility and pyocyanin production in *P. aeruginosa* (Inoue et al., 2008; Abd-Alla and Bashandy, 2012). A recent study suggested that lauric acid (C12:0) and myristic acid might interact with PsrA to enhance virulence in a murine abscess model (Juárez-Rodríguez et al., 2020). Understanding how different fatty acids modulate PsrA activity will be an important research direction for the future.

Several studies have demonstrated that PsrA plays dual regulatory roles: it activates biofilm formation, swarming motility, and T3SS genes (Kojic and Venturi, 2001; Shen et al., 2006; Gooderham et al., 2008; Kang et al., 2009), while repressing the *fadBA5* β-oxidation operon (Kang et al., 2008). Activation of *rpoS* expression was observed in the PsrA mutant in some studies (Kojic and Venturi, 2001; Shen et al., 2006) but not in others (Gooderham et al., 2008; Kang et al., 2008). Intriguingly, augmented expression of PsrA due to the addition of oleate led to decreased expression of T3SS genes and *rpoS* (Kang et al., 2009). This aligns with our transcriptomic results, indicating that PsrA can downregulate *rpoS* at higher dosages (Figure 9; Supplementary Table S3). These findings strongly suggest that PsrA’s regulatory role is highly nuanced, involving a balance between free PsrA and oleic acid-bound PsrA, additional protein factors, and bacterial growth stages. Considering the culture conditions of our study, the availability of long-chain fatty acids cannot be ruled out, complicating the assessment of free PsrA levels when only a single copy is present. Therefore, the use of a promoterless PsrA in the low-copy plasmid pMMB66EH in our study might help elucidate the role of PsrA, simulating an environment favorable for its expression.

Because of the abundance of fatty acids in living organisms, analysis of PsrA-fatty acids interaction will greatly facilitate our understanding of bacterial pathogenesis. The specificity and affinity of different fatty acids in regulating PsrA binding to the promoter of key regulatory genes can be further determined. This can be followed by studying the physiological impacts, including virulence gene expression, influenced by PsrA-fatty acid interaction. Finally, the roles of PsrA-fatty acid interaction in tissue tropism, species specificity, and competition with other microbes during *P. aeruginosa* infections will need to be elucidated.

Disabling quorum sensing has garnered interest as a strategy for treating bacterial infections. Some natural and synthetic quorum-sensing inhibitors, such as the fungi-derived component chrysophanol, can suppress quorum-sensing signals and prevent the biofilm formation of *P. aeruginosa* and *E. coli*. Another example of a natural quorum-sensing inhibitor is curcumin. When coupled with or coated onto different nanoparticles, these inhibitors exhibit a synergistic antifouling effect, eradicating biofilm formation from *P. aeruginosa* on urinary catheter surfaces (Prateeksha et al., 2019, 2021, 2023; Kumar et al., 2022). These promising approaches represent potential alternatives for the development of new antimicrobial strategies. Given PsrA’s regulation of quorum sensing and many related virulence factors, it is a viable candidate for an anti-virulence therapy target (Rémy et al., 2018; Scoffone et al., 2019).

In conclusion, we demonstrated that PsrA acted as a regulator of quorum sensing through direct regulation of *lasR*, with oleic acid acting as a crucial signaling molecule in this regulatory process. The interplay between PsrA and LasR provides a novel insight into the regulatory mechanism of quorum sensing in *P. aeruginosa* revealing the potential of PsrA as a target for therapeutic interventions.

## Data availability statement

The datasets presented in this study can be found in online repositories. The names of the repository/repositories and accession number(s) can be found in the article/Supplementary material.

## Author contributions

L-CK: Conceptualization, Data curation, Formal analysis, Investigation, Methodology, Validation, Visualization, Writing – original draft, Writing – review & editing. C-CT: Investigation, Methodology, Validation, Writing – original draft. Y-HL: Data curation, Investigation, Methodology, Writing – original draft. Y-LL: Conceptualization, Data curation, Validation, Writing – original draft. N-WC: Investigation, Writing – original draft. C-TL: Resources, Supervision, Writing – original draft, Writing – review & editing. H-YC: Conceptualization, Data curation, Funding acquisition, Project administration, Resources, Supervision, Writing – original draft, Writing – review & editing.

## Footnotes

^1^ www.pseudomonas.com
