# Supplementary material for: Roles of transcriptional factor PsrA in the regulation of quorum sensing in Pseudomonas aeruginosa PAO1
Source: Front Microbiol. 2024 Jun 26;15:1424330. doi: 10.3389/fmicb.2024.1424330 (PMC11233452; doi:10.3389/fmicb.2024.1424330)
Supplement: Supplementary file 1 [file Table_1.DOCX]

**Supplementary Table 1. Bacterial strains and plasmids used in this study.**

| **Strain/plasmid** | **Relevant characteristics** | **Sources** |
| --- | --- | --- |
| ***C. violaceum*** | | |
| CV026 | A violacein-negative, mini-Tn*5* mutant | McClean et al., 1997 |
| ***E. coli*** |  |  |
| BL21(DE3) | F^-^ *ompT gal dcm lon hsdSB(r_B_^-^ m_B_^-^) λ*(DE3[*lacI lacUV5-*T7 gene *1 ind1 sam7 nin5*]) | Laboratory stock |
| S17-1 λpir | Tp^r^ Sm^r^ *recA*, *thi*, *pro*, *hsdR^-^*M^+^ RP4:2-Tc::*Mu*:Km^r^ Tn7 λ*pir* | Laboratory stock |
| ***P. aeruginosa*** | | |
| PAO1 | Nonmucoid wild-type strain | Laboratory stock |
| Δ*psrA* | *psrA* deletion derivative of PAO1 | This study |
| Δ*lasR* | *lasR* deletion derivative of PAO1 | This study |
| Δ*psrA*Δ*lasR* | *psrA* and *lasR* deletion derivative of PAO1 | This study |
| PAO1 [pMMB66EH] | PAO1, carrying pMMB66EH, Ap^r^ | This study |
| Δ*psrA* [pMMB66EH] | Δ*psrA*, carrying pMMB66EH, Ap^r^ | This study |
| Δ*lasR* [pMMB66EH] | Δ*lasR*, carrying pMMB66EH, Ap^r^ | This study |
| Δ*psrA*Δ*lasR* [pMMB66EH] | Δ*psrA*Δ*lasR*, carrying pMMB66EH, Ap^r^ | This study |
| PAO1 [pMMB*psrA*] | PAO1, carrying pMMB*psrA*, Ap^r^ | This study |
| Δ*psrA* [pMMB*psrA*] | Δ*psrA,* carrying pMMB*psrA*, Ap^r^ | This study |
| Δ*lasR* [pMMB*psrA*] | Δ*lasR*, carrying pMMB*psrA*, Ap^r^ | This study |
| Δ*psrA*Δ*lasR* [pMMB*psrA*] | Δ*psrA*Δ*lasR*, carrying pMMB*psrA*, Ap^r^ | This study |
| **Plasmid** | | |
| pET30a | Recombinant gene expression vector, Km^r^ | Novagene |
| pET30*psrA* | pET30a with a *psrA* insert | This study |
| pEX18Tc | Tc^r^; *oriT^+^ sacB^+^*, gene replacement vector | Hoang et al., 1998 |
| pSB1075 | *lasR lasl*’::l*uxCDABE* fusion in pUC18 Amp^R^, bioluminescence producing biosensor in response to long-chain AHL | Winson et al., 1998 |
| pMMB66EH | Broad-host-range expression vector, Ap^r^ | Fürste et al., 1986 |
| pMMB*psrA* | pMMB66EH carrying a functional *psrA*, Ap^r^ | This study |
| pMMB*psrA*-FLAG | pMMB66EH carrying a functional *psrA* fused with DYKDDDDK epitope, Ap^r^ | This study |

**References**

Fürste, J.P., Pansegrau, W., Frank, R., Blöcker, H., Scholz, P., Bagdasarian, M., et al. (1986). Molecular cloning of the plasmid RP4 primase region in a multi-host-range *tacP* expression vector. *Gene* 48(1)**,** 119-131. doi: 10.1016/0378-1119(86)90358-6.

Hoang, T.T., Karkhoff-Schweizer, R.R., Kutchma, A.J., and Schweizer, H.P. (1998). A broad-host-range Flp-FRT recombination system for site-specific excision of chromosomally-located DNA sequences: application for isolation of unmarked *Pseudomonas aeruginosa* mutants. *Gene* 212(1)**,** 77-86. doi: 10.1016/s0378-1119(98)00130-9.

McClean, K.H., Winson, M.K., Fish, L., Taylor, A., Chhabra, S.R., Camara, M., et al. (1997). Quorum sensing and *Chromobacterium violaceum*: exploitation of violacein production and inhibition for the detection of *N*-acylhomoserine lactones. *Microbiology (Reading)* 143 ( Pt 12)**,** 3703-3711. doi: 10.1099/00221287-143-12-3703.

Winson, M.K., Swift, S., Fish, L., Throup, J.P., Jørgensen, F., Chhabra, S.R., et al. (1998). Construction and analysis of luxCDABE-based plasmid sensors for investigating N-acyl homoserine lactone-mediated quorum sensing. *FEMS Microbiol Lett* 163(2)**,** 185-192. doi: 10.1111/j.1574-6968.1998.tb13044.x.
