# Supplementary material for: Roles of transcriptional factor PsrA in the regulation of quorum sensing in Pseudomonas aeruginosa PAO1
Source: Front Microbiol. 2024 Jun 26;15:1424330. doi: 10.3389/fmicb.2024.1424330 (PMC11233452; doi:10.3389/fmicb.2024.1424330)
Supplement: Supplementary file 2 [file Table_2.DOCX]

**Supplementary Table 2**. Primers used in this study

| **Primer name** | **Sequence (5’→3’)** |
| --- | --- |
| **For deletion mutant** |  |
| BamHI-psrA-UF | ATTAGAATTCCGCTAAAGGCAGCGGGTTC |
| EcoRI-psrA-UR | ATAGGATCCGGTTTCTCCGCCT |
| EcoRI-psrA-DF | ATAGGGATCCTCGCAGACGGCGCCCCA |
| PstI-psrA-DR | TTATCTGCAGTCGATGCGGCTGGCGGCG |
| SacI-lasR-UF | ATGAGCTCATCCTCTGGATCAACATGGTCACCTC |
| SalI-lasR-UR | ATTGTCGACCAAGGCCATAGCGCTACGTTCTTCT |
| SalI-lasR-DF | GTCGACATCTTGCCTCTCAGGTCGGCGAG |
| HindIII-lasR-DR | AAGCTTAGTCGCTGTTCCACCAGCACTCC |
| **For gene overexpression in pMMB66EH** |  |
| EcoRI-pMMBpsrA-F | CCGGAATTCATGGCCCAGTCGGAAACCG |
| HindIII-pMMBpsrA-R | CCCAAGCTTTCAGGCCTTGGCGGGCGTCTTG |
| HindIII-pMMBpsrA-FLAG-R | CCCAAGCTTTCACTTGTCGTCATCGTCTTTGTAGTCGGCCTTGGCGGGCGTCTTG |
| Check-pMMB-F | TCAAGGCGCACTCCCGTTCTGG |
| Check-pMMB-R | CCCCACACTACCATCGGCGCTA |
| **For protein purification** |  |
| NdeI-pET30psrA-F | GGAATTCCATATGATGGCCCAGTCGGAAACCGT |
| KpnI-pET30psrA-R | GGGGTACCAAGGCCTTGGCGGGCGTCTT |
| **For qRT-PCR** |  |
| lasR-451F | CTGTGGATGCTCAAGGACTAC |
| psrA-382F | AAGTACCTGGAGGAGGTCT |
| psrA-573R | GAAGTGTTCACGCCGAAATC |
| lasR-451F | CTGTGGATGCTCAAGGACTAC |
| lasR-562R | CCACTGCAACACTTCCTTCT |
| 16S-F | CGCAACCCTTGTCCTTAGTTA |
| 16S-R | GTAAGGGCCATGATGACTTGA |
| proC-722F | TCAAATCCTTCCAGGCCAAC |
| proC-821R | TATTGGCCAAGCTGTTCGG |
| **For EMSA** |  |
| PlasR 1-210 bp-F | CCGAACTGGAAAAGTGGCTATGTC |
| PlasR 1-210 bp-R | ATGCGAACTGAATACCCAGTCACA |
| PlasR 153-344 bp-F | TGGCGATGGGCCGACAGTGA |
| PlasR 153-344 bp-R | CTATAGAGTTGGCGTTGTGCCGGA |
| PlasR 285-468 bp-F | TGCGGTCTATTGTTAAGTGGGACT |
| PlasR 285-468 bp-R | CAGCCAAATATGGATTCGGCA |
| PlasR 107-250 bp-F | CAGACGTCTGCGCCTCGGAT |
| PlasR 107-250 bp-R | AGGGTAAGCAAACGTTTAAATGTGA |
| PlasR 107-250 bp (Mutant 1)-F | CAGACGTCTGCGCCTCGGAT |
| PlasR 107-250 bp (Mutant 1)-R | AGGGTAAGCATTCGTTTAAATGTGA |
| PlasR 107-250 bp (Mutant 2)-F | CAGACGTCTGCGCCTCGGAT |
| PlasR 107-250 bp (Mutant 2)-R | AGGGTAAGCAAACGTTTAGGTGTG |
| PlasR 191-345 bp-F | ACTGGGTATTCAGTTCGCATAAAAT |
| PlasR 191-345 bp-R | TCTATAGAGTTGGCGTTGTGCCG |
| PlasR 191-345 bp (Mutant 3)-F | ACTGGGTATTCAGTTCGCATAAGGT |
| PlasR 191-345 bp (Mutant 3)-R | TCTATAGAGTTGGCGTTGTGCCG |
| PrhlR 11-293-F | TACGCGCCACTGGGAGCCTTGCTG |
| PrhlR 11-293-R | TGTGCGGTTGTGACAATTCCATGAA |
| PrhlR 258-544-F | CCTGTCGGCGTTTCATGGAATTGTC |
| PrhlR 258-544-R | CATCTCGCTACGCAAACCGTCCCA |
| PmvfR 17-280-F | TCGTTTCTTAGAACCGTTCCTGGC |
| PmvfR 17-280-R | GCGCACGGCACGGGGAGTCGTC |
| PmvfR 223-470-F | TCCTGAAACAGAGCCGTCATCCC |
| PmvfR 223-470-R | CGCTTCCCTTGATCGGGTCAG |

Underlines indicate the recognition sequence of restriction enzyme
