# Supplementary material for: Roles of transcriptional factor PsrA in the regulation of quorum sensing in Pseudomonas aeruginosa PAO1
Source: Front Microbiol. 2024 Jun 26;15:1424330. doi: 10.3389/fmicb.2024.1424330 (PMC11233452; doi:10.3389/fmicb.2024.1424330)
Supplement: Supplementary file 3 [file Table_3.DOCX]

**Supplementary Table 3**. Expression of virulence-associated genes in *P. aeruginosa* PAO1 under the effects of overexpression of PsrA

| **Gene Name** | **Locus_tag** | **Description** | | ***Fold change**  **PAO1[pMMB*psrA*]/**  **PAO1[pMMB66EH** |
| --- | --- | --- | --- | --- |
| **Type 3 secretion system component** | | | | |
| *pscP* | PA1695 | translocation protein in type III secretion | | -2.3 |
| *pscO* | PA1696 | translocation protein in type III secretion | | -2.4 |
| *PA1697* | PA1697 | type III secretion system ATPase | | -19.7 |
| *popN* | PA1698 | type III secretion outer membrane protein PopN | | -4.7 |
| *PA1699* | PA1699 | hypothetical protein | | -3.5 |
| *PA1700* | PA1700 | hypothetical protein | | -3.8 |
| *PA1701* | PA1701 | hypothetical protein | | -6.7 |
| *PA1702* | PA1702 | hypothetical protein | | -1.9 |
| *pcrD* | PA1703 | type III secretory apparatus protein PcrD | | -1.3 |
| *pcrR* | PA1704 | transcriptional regulator PcrR | | -2.5 |
| *pcrV* | PA1706 | type III secretion protein PcrV | | -6.5 |
| *pcrH* | PA1707 | regulatory protein PcrH | | -13.9 |
| *popB* | PA1708 | translocator protein PopB | | -11.0 |
| *popD* | PA1709 | translocator outer membrane protein PopD | | -10.0 |
| *exsC* | PA1710 | exoenzyme S synthesis protein ExsC | | -3.4 |
| *PA1711* | PA1711 | hypothetical protein | | -1.6 |
| *exsB* | PA1712 | exoenzyme S synthesis protein ExsB | | -4.1 |
| *exsA* | PA1713 | exoenzyme S transcriptional regulator ExsA | | -6.5 |
| *exsD* | PA1714 | hypothetical protein | | -2.1 |
| *pscB* | PA1715 | type III export apparatus protein | | -1.8 |
| *pscC* | PA1716 | type III secretion outer membrane protein PscC | | -2.5 |
| *pscD* | PA1717 | type III export protein PscD | | -1.6 |
| *pscE* | PA1718 | type III export protein PscE | | -4.3 |
| *pscG* | PA1720 | type III export protein PscG | | 5.2 |
| *pscH* | PA1721 | type III export protein PscH | | -2.0 |
| **Pyoverdine synthesis enzymes** | | | | |
| *PA2384* | PA2384 | hypothetical protein | | -8.1 |
| *pvdQ* | PA2385 | acyl-homoserine lactone acylase PvdQ | | -9.5 |
| *pvdA* | PA2386 | L-ornithine N5-oxygenase | | -12.4 |
| *fpvI* | PA2387 | RNA polymerase sigma factor | | -2.7 |
| *fpvR* | PA2388 | protein FpvR | | -2.2 |
| *pvdR* | PA2389 | pyoverdine biosynthesis protein PvdR | | -5.0 |
| *pvdT* | PA2390 | pyoverdine biosynthesis protein PvdT | | -4.1 |
| *opmQ* | PA2391 | hypothetical protein | | -2.2 |
| *pvdP* | PA2392 | pyoverdine biosynthesis protein PvdP | | -5.9 |
| *PA2393* | PA2393 | dipeptidase | | -10.1 |
| *pvdN* | PA2394 | pyoverdine biosynthesis protein PvdN | | -9.7 |
| *pvdO* | PA2395 | pyoverdine biosynthesis protein PvdO | | -5.6 |
| *pvdF* | PA2396 | pyoverdine synthetase F | | -3.7 |
| *pvdE* | PA2397 | pyoverdine biosynthesis protein PvdE | | -5.9 |
| *fpvA* | PA2398 | ferripyoverdine receptor | | -8.7 |
| *pvdD* | PA2399 | pyoverdine synthetase D | | -6.0 |
| *pvdJ* | PA2400 | pyoverdine biosynthesis protein PvdJ | | -6.0 |
| *PA2402* | PA2402 | peptide synthase | | -5.7 |
| *PA2403* | PA2403 | hypothetical protein | | -2.1 |
| *PA2404* | PA2404 | hypothetical protein | | -2.4 |
| *PA2405* | PA2405 | hypothetical protein | | -2.3 |
| *PA2406* | PA2406 | hypothetical protein | | -3.1 |
| *PA2407* | PA2407 | adhesion protein | | -4.3 |
| *PA2408* | PA2408 | ABC transporter ATP-binding protein | | -4.5 |
| *PA2409* | PA2409 | ABC transporter permease | | -2.5 |
| *PA2410* | PA2410 | hypothetical protein | | -3.5 |
| *PA2411* | PA2411 | thioesterase | | -11.3 |
| *PA2412* | PA2412 | hypothetical protein | | -11.3 |
| *pvdH* | PA2413 | diaminobutyrate--2-oxoglutarate aminotransferase | | -7.8 |
| *PA2414* | PA2414 | L-sorbosone dehydrogenase | | -3.0 |
| *PA2415* | PA2415 | hypothetical protein | | -3.8 |
| **Alginate synthesis enzymes** | | | | |
| *algD* | PA3540 | GDP-mannose 6-dehydrogenase AlgD | | -1.1 |
| *alg8* | PA3541 | glycosyltransferase alg8 | | -1.2 |
| *alg44* | PA3542 | alginate biosynthesis protein Alg44 | | -1.1 |
| *algK* | PA3543 | alginate biosynthesis protein AlgK | | 1.6 |
| *algE* | PA3544 | alginate production protein AlgE | | -18.3 |
| *algG* | PA3545 | alginate-c5-mannuronan-epimerase AlgG | | 1.5 |
| *algX* | PA3546 | alginate biosynthesis protein AlgX | | -1.7 |
| *algL* | PA3547 | alginate lyase | | -14.4 |
| *algI* | PA3548 | alginate o-acetylase AlgI | | -2.0 |
| *algJ* | PA3549 | alginate o-acetylase AlgJ | | -4.1 |
| *algF* | PA3550 | alginate o-acetyltransferase AlgF | | -1.6 |
| *algA* | PA3551 | bifunctional mannose-1-phosphate guanylyltransferase/mannose-6-phosphate isomerase | | -2.2 |
| **Lipid A modification enzymes** | | | | |
| *arnB* | PA3552 | UDP-4-amino-4-deoxy-L-arabinose--oxoglutarate aminotransferase | | -3.7 |
| *arnC* | PA3553 | undecaprenyl-phosphate 4-deoxy-4-formamido-L-arabinose transferase | | -4.7 |
| *arnA* | PA3554 | bifunctional UDP-glucuronic acid decarboxylase/UDP-4-amino-4-deoxy-L-arabinose formyltransferase | | -4.9 |
| *arnD* | PA3555 | 4-deoxy-4-formamido-L-arabinose- phosphoundecaprenol deformylase ArnD | | -7.3 |
| *arnT* | PA3556 | 4-amino-4-deoxy-L-arabinose lipid A transferase | | -4.3 |
| *arnE* | PA3557 | 4-amino-4-deoxy-L-arabinose-phosphoundecaprenol flippase subunit ArnE | | -2.2 |
| *arnF* | PA3558 | 4-amino-4-deoxy-L-arabinose-phosphoundecaprenol flippase subunit ArnF | | -6.9 |
| *PA3559* | PA3559 | nucleotide sugar dehydrogenase | | -5.5 |
| **Siderophore biosynthetic operon** | | | | |
| *fepC* | PA4158 | ferric enterobactin transporter FepC | | -4.7 |
| *fepB* | PA4159 | iron-enterobactin transporter periplasmic binding protein | | -4.0 |
| *fepD* | PA4160 | ferric enterobactin transporter FepD | | -5.4 |
| *fepG* | PA4161 | ferric enterobactin transporter FepG | | -4.0 |
| *fepG* | PA4161 | ferric enterobactin transporter FepG | | -4.0 |
| **RNA polymerase sigma factor** | | | | |
| *rpoS* | PA3622 | RNA polymerase sigma factor RpoS | | -10.7 |
| *femR* | PA1911 | sigma factor regulator FemR | | -3.0 |
| **Extracytoplasmic function sigma factor** | | | | |
| *PA1300* | PA1300 | ECF subfamily sigma-70 factor | | -14.8 |
| *PA3410* | PA3410 | ECF subfamily sigma-70 factor | | -9.2 |
| *pvdS* | PA2426 | extracytoplasmic-function sigma-70 factor | | -6.3 |
| **Transcriptional regulator** | | | | |
| *PA0056* | PA0056 | transcriptional regulator | | -14.9 |
| *pcaR* | PA0155 | transcriptional regulator PcaR | | -15.7 |
| *PA0179* | PA0179 | two-component response regulator | | -7.3 |
| *PA0191* | PA0191 | transcriptional regulator | | -4.1 |
| *toxR* | PA0707 | transcriptional regulator ToxR | | -13.1 |
| *PA1269* | PA1269 | transcriptional regulator | | -5.9 |
| *lasR* | PA1430 | transcriptional regulator LasR | | -9.3 |
| *rsaL* | PA1431 | regulatory protein RsaL | | -11 |
| *PA1570* | PA1570 | transcriptional regulator | | -5.7 |
| *pcrH* | PA1707 | regulatory protein PcrH | | -13.9 |
| *eraR* | PA1980 | response regulator EraR | | -6.2 |
| *ptxS* | PA2259 | transcriptional regulator PtxS | | -3.7 |
| *PA2334* | PA2334 | transcriptional regulator | | -4.7 |
| *PA2449* | PA2449 | transcriptional regulator | | -4.9 |
| *PA2704* | PA2704 | transcriptional regulator | | -4.1 |
| *amrZ* | PA3385 | alginate and motility regulator Z | | -4.9 |
| *nosR* | PA3391 | regulatory protein NosR | | 12.7 |
| *rhlR* | PA3477 | transcriptional regulator RhlR | | -2.6 |
| *pchR* | PA4227 | transcriptional regulator PchR | | -4.3 |
| *PA4843* | PA4843 | two-component response regulator | | -7 |
| *PA5059* | PA5059 | transcriptional regulator | | -5.3 |
| **Phenazine biosynthesis genes** | | |  |  |
| *phzH* | PA0051 | phenazine-modifying protein | | 5.5 |
| *phzA2* | PA1899 | phenazine biosynthesis protein PhzA | | -3.3 |
| *phzB2* | PA1900 | phenazine biosynthesis protein PhzB | | -4.5 |
| *phzC2* | PA1901 | phenazine biosynthesis protein PhzC | | -9.0 |
| *phzD2* | PA1902 | phenazine biosynthesis protein PhzD | | 5.6 |
| *phzE2* | PA1903 | phenazine biosynthesis protein PhzE | | 4.9 |
| *phzF2* | PA1904 | trans-2,3-dihydro-3-hydroxyanthranilate isomerase | | 6.5 |
| *phzG2* | PA1905 | pyridoxamine 5'-phosphate oxidase | | 5.7 |
| *phzM* | PA4209 | phenazine-specific methyltransferase | | 10. |
| *phzA1* | PA4210 | phenazine biosynthesis protein | | 15.5 |
| *phzB1* | PA4211 | phenazine biosynthesis protein | | 30.1 |
| *phzC1* | PA4212 | phenazine biosynthesis protein PhzC | | 5.8 |
| *phzD1* | PA4213 | phenazine biosynthesis protein PhzD | | 6.0 |
| *phzE1* | PA4214 | phenazine biosynthesis protein PhzE | | 5.4 |
| *phzF1* | PA4215 | trans-2,3-dihydro-3-hydroxyanthranilate isomerase | | 6.2 |
| *phzF1* | PA4215 | trans-2,3-dihydro-3-hydroxyanthranilate isomerase | | 6.2 |
| *phzG1* | PA4216 | pyridoxamine 5'-phosphate oxidase | | 2.5 |
| *phzS* | PA4217 | hypothetical protein | | 22.4 |
| **PQS quorum sensing-related genes** | | | |  |
| *pqsA* | PA0996 | anthranilate--CoA ligase | | 5.6 |
| *pqsB* | PA0997 | hypothetical protein | | 7.6 |
| *pqsC* | PA0998 | hypothetical protein | | 5.8 |
| *pqsD* | PA0999 | 3-oxoacyl-ACP synthase | | 6.1 |
| *pqsE* | PA1000 | thioesterase PqsE | | 7.8 |
| *phnA* | PA1001 | anthranilate synthase component I | | 4.6 |
| *phnB* | PA1002 | anthranilate synthase component II | | 3.4 |
| **Protease** | | | | |
| *lasB* | PA3724 | Elastase | | -3.6 |
| *piv* | PA4175 | Protease IV | | -3.6 |

^*^The fold change is the gene expression ratio of overexpressing strain relative to the parental strain PAO1 (PAO1[pMMB*psrA*]/PAO1[pMMB66EH]).
