# Supplementary material for: Roles of transcriptional factor PsrA in the regulation of quorum sensing in Pseudomonas aeruginosa PAO1
Source: Front Microbiol. 2024 Jun 26;15:1424330. doi: 10.3389/fmicb.2024.1424330 (PMC11233452; doi:10.3389/fmicb.2024.1424330)
Supplement: Supplementary file 4 [file Data_Sheet_1.DOCX]

Supplementary Material

Roles of transcriptional factor PsrA in the regulation of quorum sensing in *Pseudomonas aeruginosa* PAO1

Li-Ching Kok^1^, Chia-Chun Tsai^1^, Yu-Hsuan Liao^1^, Yi-Ling Lo^1^, Nai-Wei Cheng^1^, Ching-Ting Lin^2^, Hwan-You Chang^1*^

First Author*, Co-Author, Co-Author

*** Correspondence:** Hwan-You Chang: [hychang@life.nthu.edu.tw](mailto:hychang@life.nthu.edu.tw)


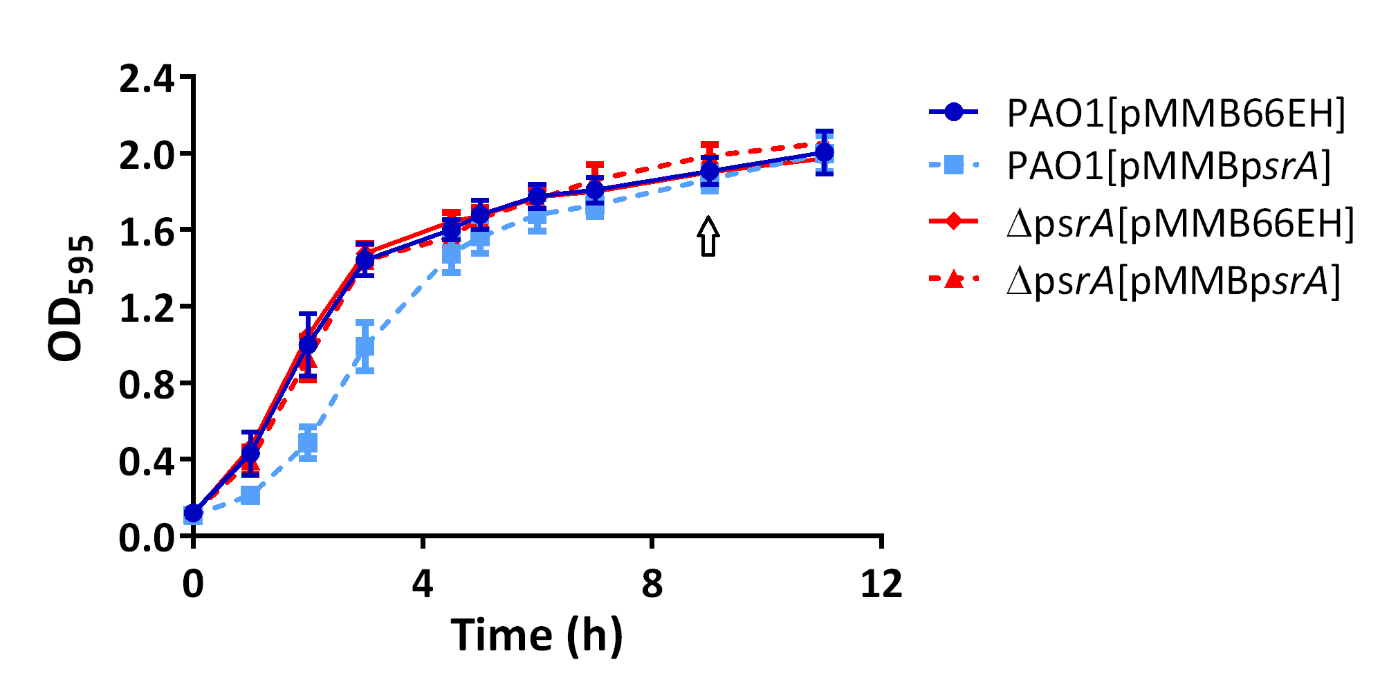


**Supplementary Figure 1.** Growth curve analysis of bacterial strains. Growth of bacterial strains was analysis in LB broth for 12 h at 37°C. The arrow indicates the optical density of cells at which (9 h) samples were collected for transcriptomic analysis. All error bars represent the standard error of the mean of triplicates.





**Supplementary Figure 2.** Western blot analysis of PsrA-FLAG. Western blot analysis of PsrA-FLAG-tagged (DYKDDDDK) proteins was conducted to assess the expression of PsrA in *P. aeruginosa* PAO1 carrying pMMB*psrA*-FLAG. Bacteria were cultured overnight with or without a final concentration of 0.01 mM IPTG or 0.05 mM IPTG to determine the minimal PsrA level that PAO1 [pMMB*psrA*-FLAG] and Δ*psrA* [pMMB*psrA*-FLAG] can express in LB without IPTG addition. Lane 1, PAO1 [pMMB66EH]; lane 2, PAO1 [pMMB*ps*r*A-*FLAG]; lane 3, Δ*psrA* [pMMB66EH]; lane 4, Δ*psrA* [pMMB*ps*r*A-*FLAG]; lane 5, PAO1 [pMMB*ps*r*A-*FLAG] induced with 0.01 IPTG; lane 6, Δ*psrA* [pMMB*ps*r*A-*FLAG] induced with 0.01 IPTG; lane 7, PAO1 [pMMB*ps*r*A-*FLAG] induced with 0.05 mM IPTG, lane 8, Δ*psrA* [pMMB*ps*r*A-*FLAG] induced with 0.05 mM IPTG.


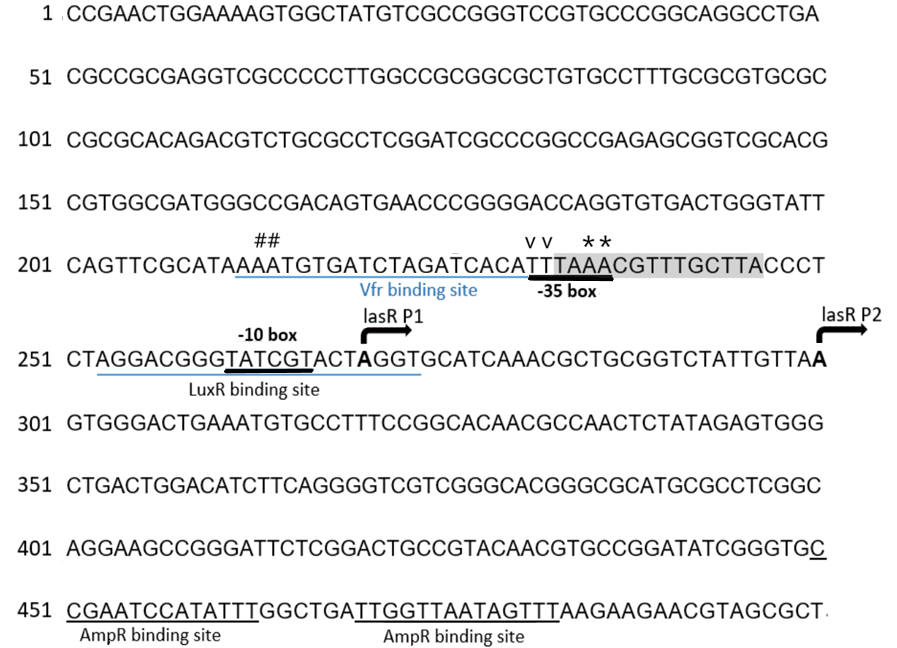


**Supplementary Figure 3**. Regulatory elements of the *lasR* promoter. The nucleotide sequence of the upstream 500-bp region of the LasR start codon. The transcriptional initiation sites for two putative *lasR* promoters are highlighted in bold and marked with an arrow. The predicted −10 and −35 sequences are marked with a thick black underline, and those predicted for Vfr, LuxR, and AmpR are indicated by a thin underline. The predicted PsrA binding site is shaded. **, ^vv^, and ^##^ indicate the site-specific mutation sites of mutant-1, mutant-2, and mutant-3 DNA fragments, respectively, used in EMSA.

(A)


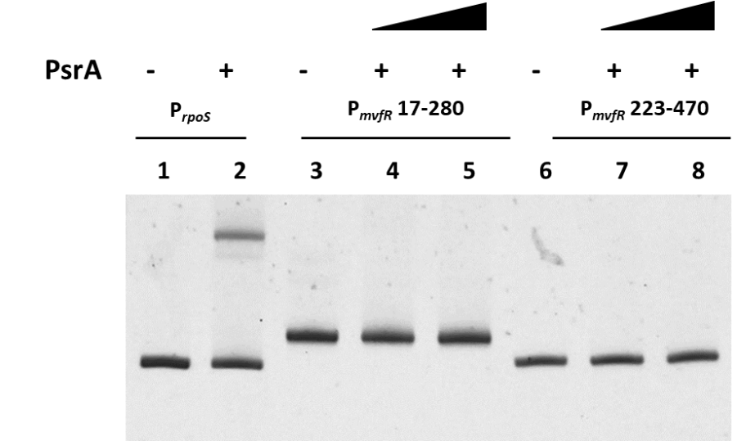


(B)


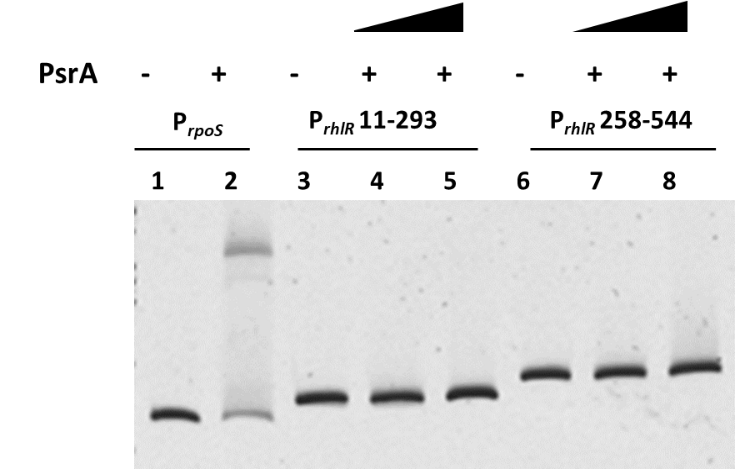


**Supplementary Figure 4.** Recognition of PsrA binding on the promoter region of *mvfR* and *rhlR* using electrophoretic mobility shift assay. The DNA fragments used in the study areas are as follows: **(A)** Lanes 1, 2, *P_rpoS_* as a positive control; lanes 3, 4, 5, *P_mvfR_*17–280 bp; lane 6, 7, 8, *P_mvfR_*223–470 bp. Lanes 1, 3, and 6 are loaded with DNA fragments, while lanes 2, 4, 5, 7, and 8 are loaded with a mixture of DNA fragments and PsrA.  **(B)** Lane 1, 2, *P_rpoS_* as a positive control; lane 3, 4, 5, *P_rhlR_*11–280 bp; lane 6, 7, 8, *P_rhlR_*258–544 bp. Lanes 1, 3, and 6 are loaded with DNA fragments, while lanes 2, 4, 5, 7, and 8 are loaded with a mixture of DNA fragments and PsrA. In each reaction, 100 ng of DNA was used, while 0.5 and 1 µg of PsrA was used for Lanes 4, 5, and Lane 2, 7, and 8, respectively.
